# A novel molecular-clinicopathologic nomogram to improve prognosis prediction of hepatocellular carcinoma

**DOI:** 10.18632/aging.103350

**Published:** 2020-06-30

**Authors:** Zhongjing Zhang, Wanqing Weng, Weiguo Huang, Boda Wu, Yi Zhou, Jie Zhang, Tuo Deng, Wen Ye, Jiecheng Zhang, Jianyang Ao, Qiyu Zhang, Keqing Shi

**Affiliations:** 1 Department of Hepatopancreatobiliary Surgery, The First Affiliated Hospital, Wenzhou Medical University, Wenzhou 325015, Zhejiang Province, PR China; 2 Precision Medical Center Laboratory, The First Affiliated Hospital, Wenzhou Medical University, Wenzhou 325015, Zhejiang Province, PR China

**Keywords:** hepatocellular carcinoma, the cancer genome atlas, long non-coding RNA, nomogram, time-dependent receiver operating characteristic

## Abstract

Background: Emerging evidence suggests that long non-coding RNA (lncRNA) plays a crucial part in the development and progress of hepatocellular carcinoma (HCC). The objective was to develop novel molecular-clinicopathological prediction methods for overall survival (OS) and recurrence of HCC.

Results: An 8-lncRNA-based classifier for OS and a 14-lncRNA-based classifier for recurrence were developed by LASSO COX regression analysis, both of which had high accuracy. The tdROC of OS-nomogram and recurrence-nomogram indicates the satisfactory accuracy and predictive power. The classifiers and nomograms for predicting OS and recurrence of HCC were validated in the Test and GEO cohorts.

Conclusions: These two lncRNA-based classifiers could be independent prognostic factors for OS and recurrence. The molecule-clinicopathological nomograms based on the classifiers could increase the prognostic value.

Methods: HCC lncRNA expression profiles from the cancer genome atlas (TCGA) were randomly divided into 1:1 training and test cohorts. Based on least absolute shrinkage and selection operator method (LASSO) COX regression model, lncRNA-based classifiers were established to predict OS and recurrence, respectively. OS-nomogram and recurrence-nomogram were developed by combining lncRNA-based classifiers and clinicopathological characterization to predict OS and recurrence, respectively. The prognostic value was accessed by the time-dependent receiver operating characteristic (tdROC) and the concordance index (C-index).

## INTRODUCTION

Hepatocellular carcinoma (HCC) is one of the leading causes of cancer-related mortality worldwide [1]. Considerable progress has been achieved in the prevention, monitoring, early screening, diagnosis and treatment of HCC over the past few decades. However, in many countries, the incidence and specific mortality of HCC continue to rise [2]. There are a number of reasons for the high mortality rate of HCC; most importantly, in many parts of the world, patients are diagnosed at an advanced stage [1]. Thence, it is of great clinical implication to identify effective tumor markers and explore their role in the occurrence and development of HCC.

Next-generation sequencing (NGS) is a powerful platform for high-throughput sequencing of different genetic factors, which helps researchers to obtain more accurate and comprehensive data of gene variation [3]. The rich and standardized clinical data and abundant samples for different types of cancer generated by the Cancer Genome Atlas (TCGA) enabled a joint analysis of multiple influencing factors associated with tumor oncogenesis [4, 5].

Long non-coding RNAs (lncRNAs) which contain more than 200 nucleotides is a type of the non-coding RNAs (ncRNAs) [6]. For a long time, lncRNA is considered as a kind of non-functional RNA, but emerging research has proved that these RNAs were important regulators of gene expression networks [7, 8]. Their functions contain controlling nuclear architecture and mRNA stability, participating in the transcription, translation and post-translational modifications, which involve all aspects of cellular gene expression [9, 10]. In recent researches, many lncRNAs have seemed as biomarkers of early detection and prognosis of HCC, but these studies only involved minority lncRNA and lack a large number of clinical samples for analysis [11, 12].

In current study, we collected a large cohort of HCC patients who contained clinical information and complete sequencing results in the TCGA database. Thereafter, we performed least absolute shrinkage and selection operator method (LASSO) COX select model, a method that could be applied to high dimensional regression prediction, to establish and validate two multi-lncRNA-based classifiers which have high veracity of predicting overall survival (OS) and recurrence in HCC patients.

## RESULTS

### Data processing

**Figure 1:**
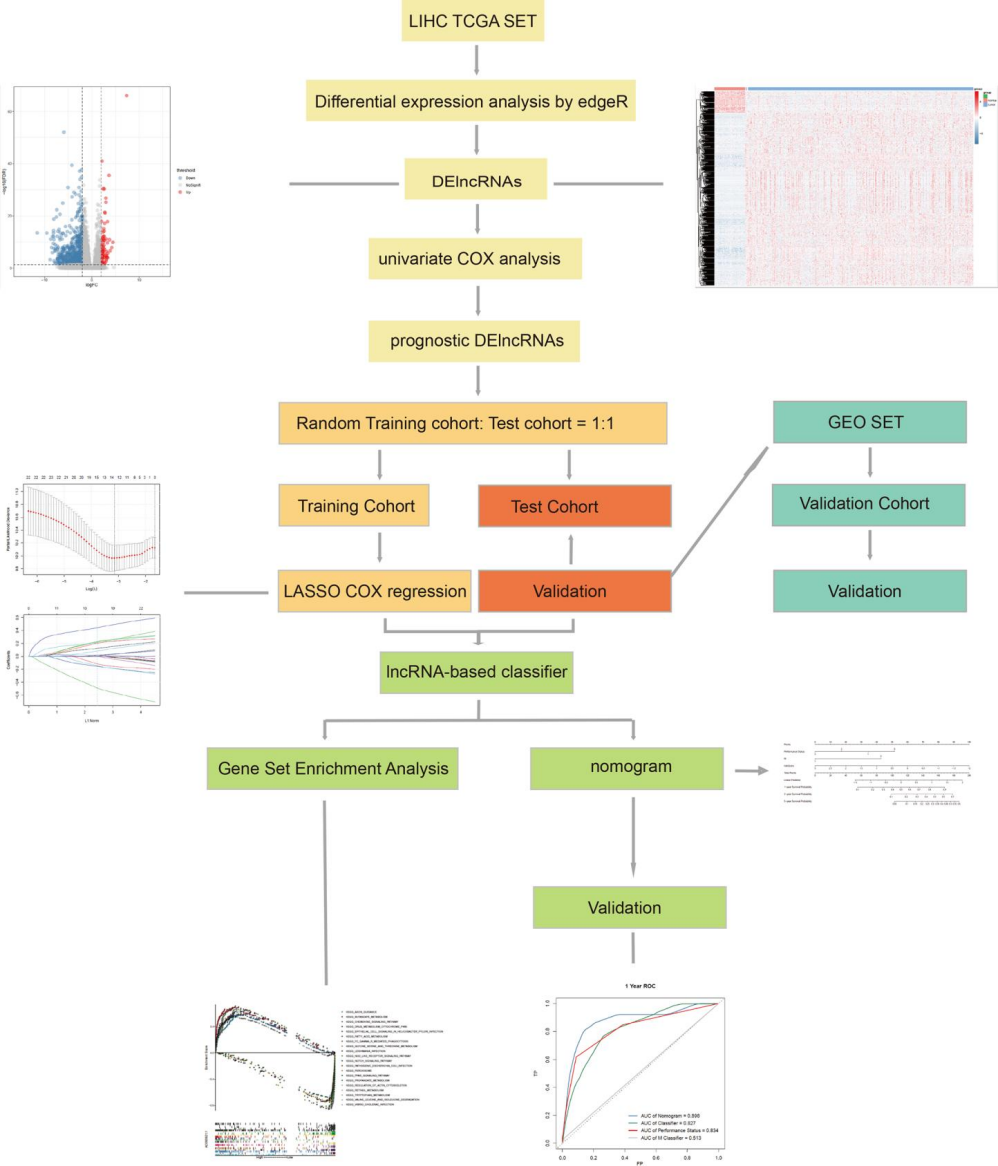
**The workflow of this work.**

**Figure 2:**
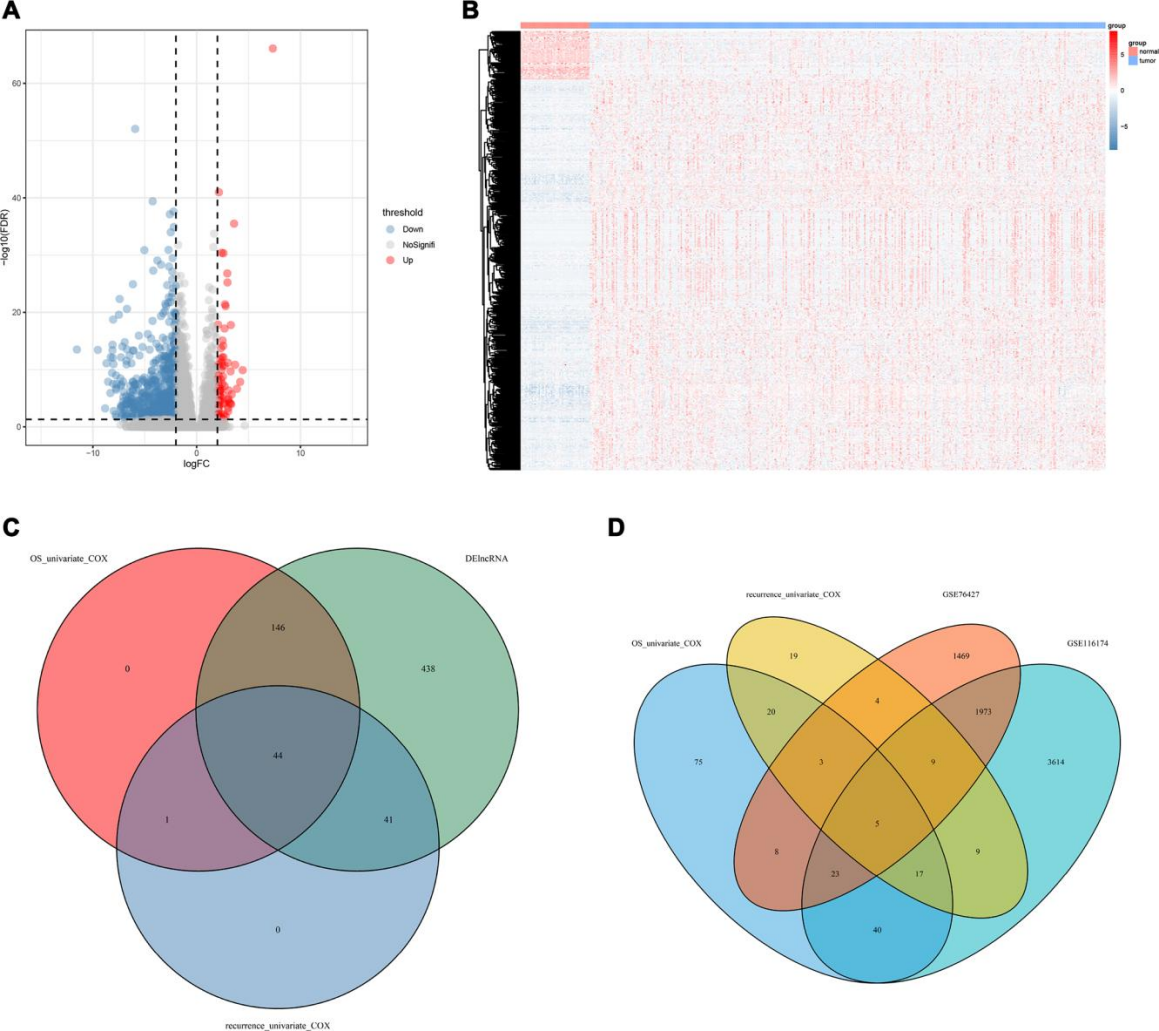
**Prognostic DElncRNAs identification process.** (**A**) Volcano plot of differentially expressed lncRNAs in TCGA-LICH dataset; (**B**) Hierarchical clustering of HCC with or without tumor using 669 differentially expressed lncRNAs using Euclidean distance and average linkage clustering; (**C**) Venn diagram of prognostic DElncRNAs in prognostic lncRNAs (OS/recurrence multivariate cox p < 0.05) and DElncRNAs(|logFC| >2 and padj < 0.05); (**D**) Venn diagram of lncRNAs related to OS/recurrence. TCGA, The Cancer Genome Atlas; LICH, Liver hepatocellular carcinoma; HCC, hepatocellular carcinoma; DElncRNA, differentially expressed long non-coding RNA; OS, overall survival; LASSO, least absolute shrinkage and selection operator method.

**Figure 3:**
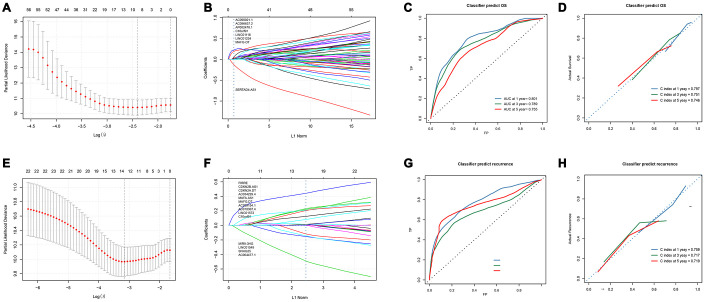
**Development and verification of 8-lncRNAs-based and 14-lncRNAs-based classifiers.** (**A**) LASSO coefficient profiles of the 86 Significant difference lncRNAs in OS set. A vertical line is drawn at the value chosen by 10-fold cross-validation; (**B**) Ten-time cross-validation for tuning parameter selection in the LASSO model; (**C**, **D**) Time-dependent ROC curves and calibration curves of 8-lncRNAs-based classifier; (**E**) Time-dependent ROC curves of Liao’s biomarker for overall survival; (**F**) LASSO coefficient profiles of the 21 Significant difference lncRNAs in recurrence set, A vertical line is drawn at the value chosen by 10-fold cross-validation; (**G**) Ten-time cross-validation for tuning parameter selection in the LASSO model; (**H**) Time-dependent ROC curves and calibration curves of 14-lncRNAs-based classifier. LASSO, least absolute shrinkage and selection operator method; lncRNA, long non-coding RNA; OS, overall survival; ROC, receiver operating characteristic.

The workflow of this article is shown in Figure 1. In the expression profiles of HCC tumors compared with the samples from normal tissues, we identified 669 differentially expressed lncRNAs (DElncNRAs) of |log Fold Change| ≥ 2 and p < 0.05(Supplementary Table 1 and Figure 2A). Of which, 595 lncRNAs were down-regulated and 74 lncRNAs were up-regulated. As shown in Figure 2B, significant differential expression was detected between the tumor and the adjacent normal groups. Subsequently, the DElncRNAs with P <0.05 were selected by univariate COX regression analysis. Therefore, a total of 191 OS-related lncRNAs and 86 recurrence-related lncRNAs were reserved for further study (Figure 2C). After taking the intersection with GSE76427 and GSE116174, 21 recurrence-related lncRNAs and 85 OS-related lncRNAs were finally obtained for classifier development. A total of 312 patients with OS data were randomized 1:1 into two groups, the training cohort (n=156) and the test cohort (n=156). The GSE116174 (n=64) was reserved as a validation cohort for predicting OS. Meanwhile, a total of 269 patients with recurrence data were randomly divided equally into two groups, the training cohort (n= 130) and the test cohort (n=139). GSE76427 (n= 81) was used as a validation cohort to validate recurrence-related models. LASSO COX selection method was applied to training cohort to develop a prediction model (OS: Figure 3A, 3B; recurrence: 3F, 3G). As shown in Supplementary Tables 2, 8 OS-related DElncRNAs and 14 recurrence-related DElncRNAs were identified by the LASSO COX selected model.

### Multi-lncRNAs-based classifier

In order to contrive multi-lncRNAs-based classifiers for predicting OS and recurrence in HCC, LASSO COX selection method was performed with the 85 OS related lncRNAs and 21 recurrence related lncRNAs expression data. An 8-lncRNA-based classifier for OS (Figure 3A and 3B) and a 14-lncRNAs-based classifier for recurrence were constructed by training cohort (Figure 3E, 3F). All those lncRNAs are listed in Supplementary Table 2. 8-lncRNAs-based classifier = 0.0299*EXP (AC090921.1) + 0.0125*EXP (AC096637.2) + 0.1838*EXP(AP002478.1) + 0.2221*EXP (C10orf91)+ 0.0437*EXP (LINC01116) + 0.0251*EXP (LINC01224) + 0.0137*EXP (MAFG-DT) -0.1168*EXP(SERTAD4-AS1); 14-lncRNAs-based classifier = -0.0255*EXP (AC004477.1)+0.1647*EXP(AC010307.4)+0.0416*EXP(AC034229.4)+0.1580*EXP (AC209154.1)+0.3958*EXP(C10orf91)+0.0233*EXP(CDKN2A-DT)+0.0037*EXP(CDKN2B-AS1)+0.00057*EXP(FIRRE)-0.1140*EXP(LINC01549)+0.1813*EXP (LINC01572)+0.0958*EXP(MAFA-AS1)+ 0.1348*EXP(MAFG-DT)-0.365*EXP (MIR9-3HG)-0.0761*EXP (SNHG25). All patients were divided into low and high risk groups according to the optimal cut-off value calculated by X-TILE. The optimal cutoff value for the OS-related classifier was 0.2, and for the recurrence-related classifier was 0.1. The Kaplan-Meier log rank test illustrated that there were significant differences in OS and recurrence in the training cohort (Supplementary Figure 1A, 1E), the test cohort (Supplementary Figure 1B, 1F), the TCGA cohort (Supplementary Figure 1C, 1G), and the GEO cohort (Supplementary Figure 1D, 1H).

### Patient characteristics

Since the training cohort and test cohort were equally randomly grouped, there was no significant difference or deviation between them. (Supplementary Tables 9–11, Table 1).

**Table 1:** Univariate and multivariate COX analyses of the lncRNA-based classifier for recurrence.

**Prognostic parameter**	**Univariate analysis**				**Multivariate analysis**		
	**HR**	**95% CI**	**P value**		**HR**	**95% CI**	**P value**
**Training Cohort**							
RiskScore	4.434	2.860-6.874	**0.001**		6.210	3.092-12.474	**0.001**
Age	0.980	0.613-1.566	0.932				
M	5.661	1.325-24.179	**0.019**		1.957	0.388-9.871	0.416
N	6.040	0.794-45.943	0.082				
Stage	2.392	1.364-4.196	**0.002**		4.872	2.023-11.732	**0.001**
T classification	2.486	1.469-4.207	**0.001**				
Bilirubin	1.140	0.908-1.430	0.259				
Child-Pugh classification	0.744	0.262-2.111	0.579				
Performance Status	1.766	1.276-2.445	**0.001**		1.119	0.740-1.692	0.595
Family History	0.851	0.508-1.428	0.542				
Fraction Genome Altered	2.187	0.671-7.133	0.194				
Grade	0.650	0.383-1.104	0.111				
Adjacent hepatic tissue inflammation	1.098	0.704-1.712	0.681				
HBV	0.403	0.230-0.705	**0.001**		0.782	0.402-1.984	0.782
HCV	1.463	0.845-2.530	0.173				
Alcohol	1.063	0.639-1.769	0.814				
Liver fibrosis Ishak score category	1.077	0.887-1.308	0.453				
Mutation Count	1.001	1.000-1.002	0.085				
Platelet count	1.000	1.000-1.000	0.452				
Race Category	1.123	0.893-1.413	0.320				
Albumin	1.067	1.014-1.123	**0.013**		1.008	0.953-1.065	0.792
Gender	1.252	0.745-2.103	0.386				
Vascular Invasion	0.785	0.509-1.211	0.273		0.536	0.291-0.987	**0.045**
BMI	0.979	0.936-1.024	0.363				
AFP	0.992	0.938-1.050	0.792				
**Test Cohort**							
RiskScore	1.448	1.060-1.977	**0.020**		1.448	0.903-2.324	0.125
Age	0.829	0.520-1.320	0.430				
M	1.000		1.000				
N	0.845	0.117-6.134	0.868				
Stage	0.001	1.530-3.945	**0.001**		0.658	0.051-8.531	0.749
T classification	2.232	1.405-3.547	**0.001**		2.311	0.196-27.305	0.506
Bilirubin	1.045	0.948-1.152	0.379				
Child-Pugh classification	3.187	1.218-8.338	**0.018**		2.516	0.636-9.949	0.188
Performance Status	1.922	1.471-2.513	**0.001**		1.494	0.802-2.784	0.206
Family History	0.929	0.566-1.526	0.772				
Fraction Genome Altered	2.598	0.808-8.350	0.109				
Grade	1.509	0.960-2.373	0.075				
Adjacent hepatic tissue inflammation	1.386	0.880-2.181	0.159				
HBV	0.537	0.314-0.918	**0.023**		0.793	0.395-1.592	0.514
HCV	1.578	0.864-2.882	0.138				
Alcohol	1.104	0.700-1.742	0.670				
Liver fibrosis Ishak score category	0.989	0.842-1.161	0.891				
Mutation Count	1.002	1.000-1.004	0.118				
Platelet count	1.000	1.000-1.000	0.260				
Race Category	0.995	0.785-1.259	0.964				
Albumin	0.986	0.937-1.038	0.592				
Gender	0.962	0.612-1.512	0.866				
Vascular Invasion	1.101	0.739-1.640	0.637		0.953	0.516-1.761	0.878
BMI	1.004	0.982-1.027	0.712				
AFP	1.042	0.988-1.097	0.127				
**TCGA Cohort**							
RiskScore	2.065	1.625-2.626	**0.001**		2.043	1.458-2.863	**<0.001**
Age	0.903	0.650-1.255	0.543				
M	7.067	2.197-22.730	**0.001**		7.520	2.250-25.14	**0.001**
N	1.648	0.405-6.701	0.485				
Stage	2.405	1.683-3.435	**0.001**				
T classification	2.320	1.643-3.277	**0.001**		1.646	0.374-7.247	0.510
Bilirubin	1.060	0.974-1.153	0.180				
Child-Pugh classification	1.323	0.658-2.661	0.433				
Performance Status	1.863	1.520-2.283	**0.001**		1.770	1. 376-2.276	**<0.001**
Family History	0.901	0.630-1.288	0.568				
Fraction Genome Altered	2.356	1.026-5.411	**0.043**		1.177	0.368-3.765	0.784
Grade	1.034	0.740-1.445	0.845				
Adjacent hepatic tissue inflammation	1.222	0.891-1.676	0.213				
HBV	0.457	0.311-0.671	**0.001**		0.849	0.510-1.414	0.530
HCV	1.453	0.971-2.172	0.069				
Alcohol	1.074	0.768-1.504	0.676				
Liver fibrosis Ishak score category	1.028	0.908-1.164	0.660				
Mutation Count	1.001	1.000-1.002	**0.031**				
Platelet count	1.000	1.000-1.000	0.189				
Race Category	1.054	0.895-1.241	0.530				
Albumin	0.999	0.994-1.004	0.700				
Gender	1.078	0.769-1.512	0.663				
Vascular Invasion	0.908	0.677-1.217	0.517		0.951	0.656-1.397	0.790
BMI	0.998	0.975-1.021	0.853				
AFP	1.016	0.978-1.056	0.403				
**GSE76427 Cohort**							
RiskScore	1.433	0.920-2.232	0.112				
Age	1.069	0.586-1.951	0.828				
Gender	0.613	0.269-1.394	0.243				
Stage	1.279	0.608-2.693	0.517				

HR, Hazard ratio; CI, confidence interval; lncRNA, long non-coding RNA.

### Overall survival

In the training cohort, 156 patients were enrolled. As shown in Supplementary Table 9, there were no significant differences were detected in the distribution of age (P = 0.598), neoplasm histologic grade (P = 0.179), vascular invasion (P = 0.872), performance status (P = 0.155), TNM T stage (P=0.523), TNM M stage (P = 0.298), adjacent hepatic tissue inflammation (P = 0.656), liver fibrosis Ishak score category (P = 0.923), family history (P = 0.922), race category (P = 0.968), HBV infection (P = 0.080), HCV infection (P = 0.139), alcohol consumption (P = 0.287), Child-Pugh classification (P = 0.068), AJCC pathological stage (P = 0.521) and gender (P = 0.849).

In the test cohort, the distribution of data was similar to the training cohort. The proportion of patients with performance status (2 + 3) (P = 0.018), TNM N stage (N1) (P = 0.031) and neoplasm histologic Grade (G3+G4) (P = 0.027) in the high-risk group was higher than the low risk group.

In the TCGA cohort, 312 patients were included for further study. The proportion of patients with performance status (2 + 3) (P = 0.018), tumor grade (G3 + G4) (P = 0.012), and HBV infection (P = 0.043) in the high-risk group was higher than the low risk group.

In the GSE116174 cohort, 64 patients were enrolled. As shown in Supplementary Table 9, there were no significant differences were detected in the distribution of age (P = 0.516), gender (P = 0.418), vascular invasion (P = 0.612), HBV infection (P = 0.849), HCV infection (P = 0.139), Alcohol consumption (P = 0.167), and AJCC pathological stage (P = 0.754).

As shown in Supplementary Figure 2A–2D), the AJCC pathological stage, performance status, HBV infection and neoplasm histologic grade are significantly correlated with the 8-lncRNAs-based classifier. The 8-lncRNAs-based classifier scores for the performance status (2 & 3 & 4), stage (III & IV), HBV positive, and tumor grade (G3 & G4) groups were higher than those of the performance status (0 & 1), stage (I & II), HBV negative, and tumor grade (G1 & G2) groups.

### Recurrence

In the training cohort, 130 patients were enrolled. As shown in Supplementary Table 10, the proportion of patients with performance status (2 + 3) (P=0.026), Child-Pugh classification (C&D) (P=0.030), TNM T stage (T3 + T4) (P=0.006), and AJCC pathological stage (III & IV) (P=0.023) in the high-risk group was higher than the low risk group.

In the test cohort, the proportion of patients with tumor grade (G3 + G4) (P=0.006) in the high-risk group was higher than the low risk group. The remaining risk factors were not significantly different in distribution compared to the training cohort (Supplementary Table 10).

In the TCGA cohort, a total 269 patients were enrolled. As shown in Supplementary Table 10, The proportion of patients with tumor grade (G3 + G4) (P = 0.005), HBV infection (P = 0.001), TNM T stage (T3 + T4) (P = 0.029), TNM N stage (N1) (P = 0.023), and AJCC pathological stage (III & IV) (P = 0.007) in the high-risk group was higher than the low risk group.

In the GSE76427 cohort, 81 patients were enrolled. As shown in Supplementary Table 10, there were no significant differences were detected in the distribution of age (P = 0.960), AJCC pathological stage (P = 0.303) and gender (P = 0.117).

As shown in Supplementary Figure 2A–2D), the AJCC pathological stage, performance status, HBV infection and neoplasm histologic grade were significantly correlated with the 14-lncRNAs-based classifier. The 14-lncRNAs-based classifier scores for the performance status (2 & 3), stage (III & IV), HBV positive, and tumor grade (G3 & G4) groups were higher than those of the performance status (0 & 1), stage (I & II), HBV negative, and tumor grade (G1 & G2) groups. In addition, we also investigated the relationship between a total of 20 lncRNAs. The results are shown in Supplementary Table 9.

### Prognosis value of the lncRNA-based classifiers

Additionally, we assessed the prognostic value of lncRNA-based classifiers.

### Overall survival

Cox univariate analysis showed that the Performance Status, the tumor stage, TNM T classification, HBV infection, and the 8-lncRNA-based classifier were correlated with OS, whether in the training cohort, test cohort, or the TCGA cohort. After multivariable adjustment by these variables, Performance Status (HR: 2.589, 95% CI: 1.355-4.947; P = 0.004), TNM M stage (HR: 7.703, 95% CI: 1.603-37.021; P = 0.011), and the lncRNA-based classifier (HR: 15.483, 95% CI: 6.149-38.989; P < 0.001) remained to be powerful and independent factors for OS in the TCGA Cohort (Supplementary Table 11). In addition, multiple lncRNAs-based classifier was still an independent risk factor in the validation cohort (GSE116174).

In the time-dependent ROC curve, the 8-lncRNAs-based classifiers can effectively predict the 1-year, 3-year, and 5-year survival rates, and their AUC is 0.801, 0.789 and 0.755, respectively (Figure 3C). The average predicted probability (predicted survival rate) and Kaplan-Meier estimated (observed survival rate) were plotted, and the dotted line indicated the ideal reference line corresponding to the predicted survival rate and the actual survival rate. The calibration curve of 1-, 3- and 5-year survival probability based on 8-lncRNAs-classifier were in good agreement with the actual observed values. The C-index of 1-year, 3-year, and 5-year were 0.797, 0.751 and 0.746 respectively, indicating that the prediction model had high accuracy (Figure 3D). Compared with tdROC of liao et al., [13] a larger AUC indicated that our model had a good prediction ability (Supplementary Figure 4A–4C).

### Recurrence

Cox univariate analysis showed that Performance Status, the tumor stage, TNM T stage, TNM M stage, HBV infection, and the 14-lncRNA-based classifier were correlated with recurrence, whether in the training cohort, test cohort, or the TCGA cohort. After multivariable adjustment by these variables, Performance Status (HR: 1.608, 95% CI: 1.213-2.131; P < 0.001), TNM M stage (HR: 5.782, 95% CI: 1.631-20.501; P = 0.007) and the lncRNA-based classifier (HR: 2.076, 95% CI: 1.457-2.957; P < 0.001) remained to be powerful and independent factors for recurrence in the TCGA cohort (Table 1).

In the time-dependent ROC curve, the 14-lncRNAs-based classifiers can effectively predict the 1-year, 3-year, and 5-year survival rates with AUC of 0.800, 0.686 and 0.789, respectively (Figure 3G). The average predicted probability (predicted survival rate) and Kaplan-Meier estimated (observed survival rate) were plotted, and the dotted line indicated the ideal reference line corresponding to the predicted survival rate and the actual survival rate. The calibration curve of 1-, 3- and 5-year survival probability based on 14-lncRNAs-classifier are in good agreement with the actual observed values. The C-index of 1-year, 3-year, and 5-year were 0.759, 0.717 and 0.719 respectively, indicating that the prediction model had good performance (Figure 3H). Compared with tdROC of liao et al., [13] a larger AUC indicated that our model had a good prediction ability. (Supplementary Figure 4D–4F)

### Construction and evaluation of the nomogram

**Figure 4:**
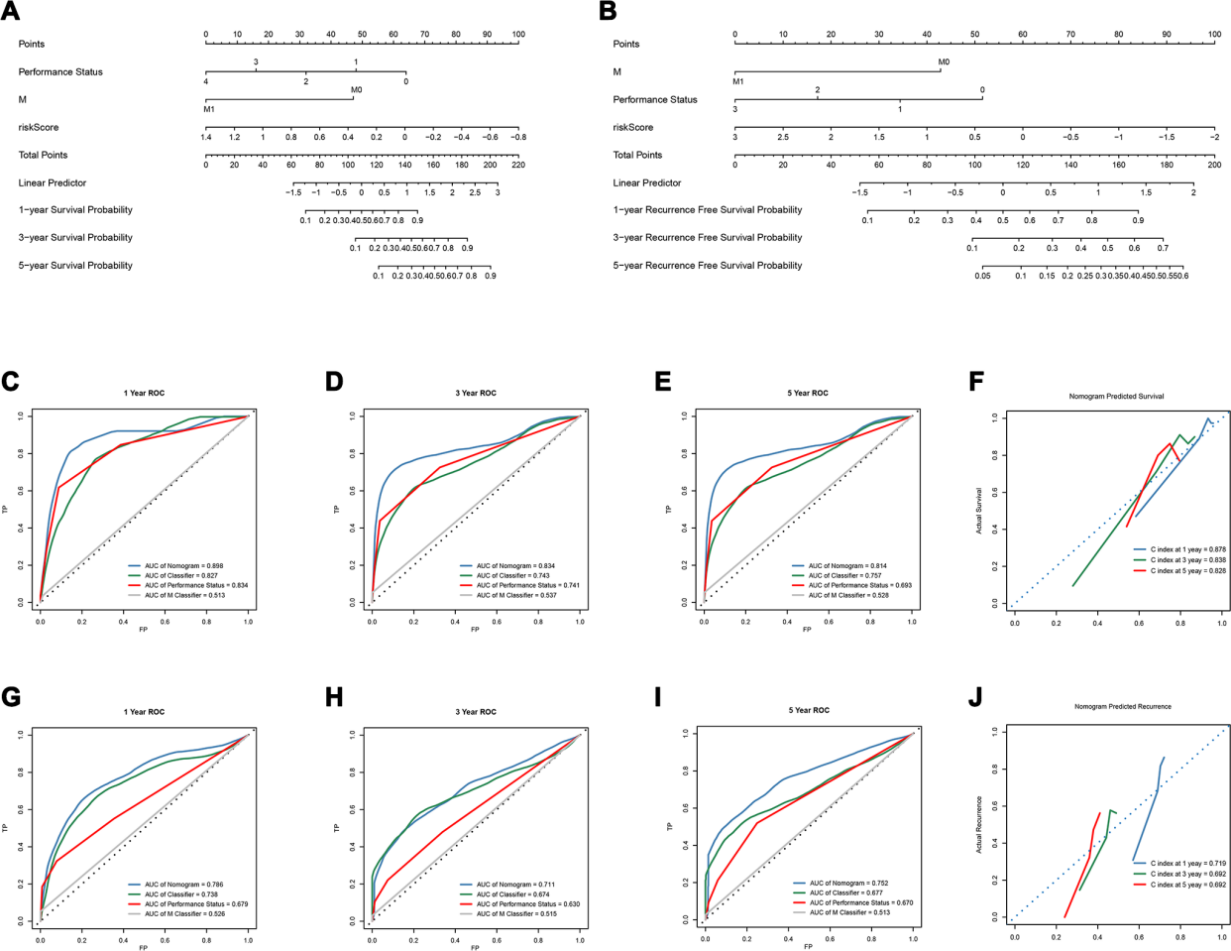
**Development and verification of OS-nomogram and recurrence-nomogram.** (**A**) OS-nomogram based on 8-lncRNAs-based classifier, TNM M classifier and Performance Status; (**B**) recurrence-nomogram based on 14-lncRNAs-based classifier, TNM M classifier and Performance Status; (**C**–**E**) The 1, 3, and 5-year Time-dependent ROC curves compare the prognostic accuracy of the OS-nomogram; (**F**) 1, 3, and 5 year calibration curve and C-index of the OS-nomogram; (**G**–**I**) The 1, 3, and 5-year Time-dependent ROC curves compare the prognostic accuracy of the recurrence-nomogram; (**J**) 1, 3, and 5 year calibration curve and C-index of the recurrence-nomogram. OS, overall survival; lncRNA, long non-coding RNA; ROC, receiver operating characteristic; C-index, concordance index.

Subsequently, we constructed a gene-clinical nomogram (Figure 4A, 4B) by multivariate cox regression analysis (Supplementary Table 11, Table 1), combined with clinical characterization and lncRNAs-based classifier. TNM M stage, Performance Status and an 8-lncRNAs-based classifier were included in the gene-clinical nomogram of OS, while TNM M stage, Performance Status and 14-lncRNAs-based classifier were included in the gene-clinical nomogram of recurrence. Nomograms can visually predict the prognosis of patients according to their genes and clinical information, and accurately predict the survival and recurrence of patients at 1-, 3-, and 5 years. Moreover, the score of the nomogram was retained for development and validation of the performance of the nomogram risk score (Supplementary Tables 3 and 4). The risk score of OS-nomogram = 1/(-45.629*Classifier - 47*M - 16*Performance Status + 134.689). The risk score of recurrence-nomogram = 1/(-20*Classifier - 43*M -17.3*Performance Status + 94.7). And the accuracy of the nomogram in 1, 3 and 5 years was analyzed by tdROC, and the corresponding calibration curve was drawn (Figure 4).

### Overall survival

The OS-nomogram based on an 8-lncRNAs-based classifier combined with the TNM M stage and Performance Status has an AUC of 0.898, 0.834, and 0.814 for predicting 1, 3, and 5 years of OS, respectively. The C-index of 1, 3, and 5 years was 0.878, 0.838, and 0.828, respectively (Figure 4C–4F). The results indicated that the combination of the lncRNA-based classifier models, TNM M stage and Performance Status could enhance the capability to predict the prognosis of survival. Kaplan-Meier curve analysis showed that the two groups divided by cutoff value (0.006953) calculated by X-tile were still statistically significant in OS (Supplementary Figure 3A–3C).

**Figure 5:**
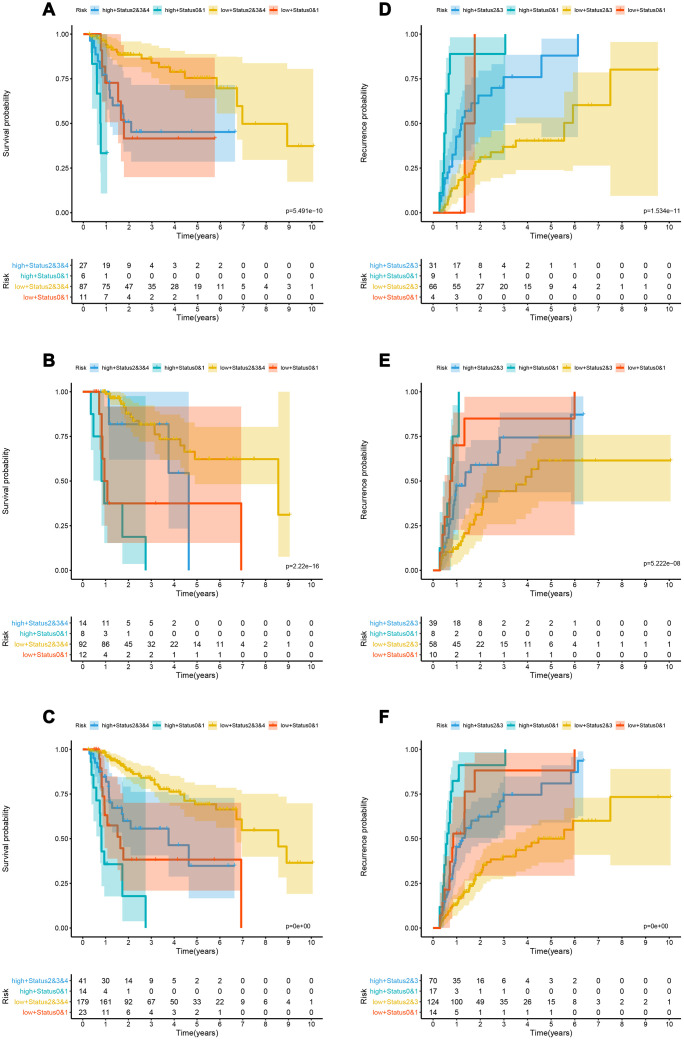
**Kaplan-Meier analysis in the training, validation and whole cohorts according to the lncRNA-based classifiers stratified by clinicopathological risk factors.** (**A**–**C**) Kaplan-Meier survival curves of LIHC patients with combinations of lncRNA-classifier and TNM T classifier in the training, test and TCGA cohorts for OS; (**D**–**F**) Kaplan-Meier survival curves of LIHC patients with combinations of lncRNA-classifier and TNM stage in the training, test and TCGA cohorts for OS. lncRNA, long non-coding RNA; OS, overall survival; LIHC, Liver hepatocellular carcinoma.

Kaplan-Meier curve showed that patients in the training cohort, the test cohort and the TCGA cohort distributed by lncRNA-based classifiers with Performance Status had significantly different prognosis (p < 0.0001, Figure 5A–5C). As shown in Supplementary Table 5, the tests were performed by log rank test between groups.

### Recurrence

The td-ROC showed that the recurrence-nomogram based on classifier, TNM M stage and Performance Status has an AUC of 0.786, 0.711, and 0.752 for predicting 1, 3, and 5 years of recurrence, respectively. The C-index of 1, 3, and 5 years was 0.719, 0.692, and 0.692, respectively (Figure 4G–4J). The Kaplan-Meier curve analysis also indicated that the prognosis of patients stratified by cutoff value (0.01470) calculated by X-tile was significantly different (Supplementary Figure 3D–3F). The lncRNA-based classifiers with TNM stage could distinguish patients in the training cohort, the test cohort and the TCGA cohort into the different risk of recurrence (p< 0.0001, Figure 5D–5F). As shown in Supplementary Table 6, the tests were performed by log rank test between groups.

### GSEA identifies KEGG signaling pathway

**Figure 6:**
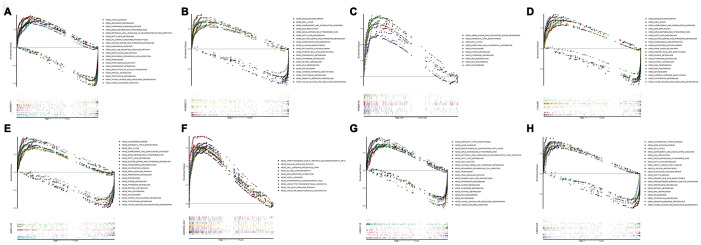
**Gene set enrichment analysis of lncRNAs of overall survival related classifier.** GSEA results showed in (**A**) AC090921.1, (**B**) AC096637.2, (**C**) AP002478.1, (**D**) C10orf91, (**E**) MAFG−DT, (**F**) SERTAD4−AS1, (**G**) LINC01116, and (**H**) LINC01224. GSEA, Gene set enrichment analysis.

**Figure 7:**
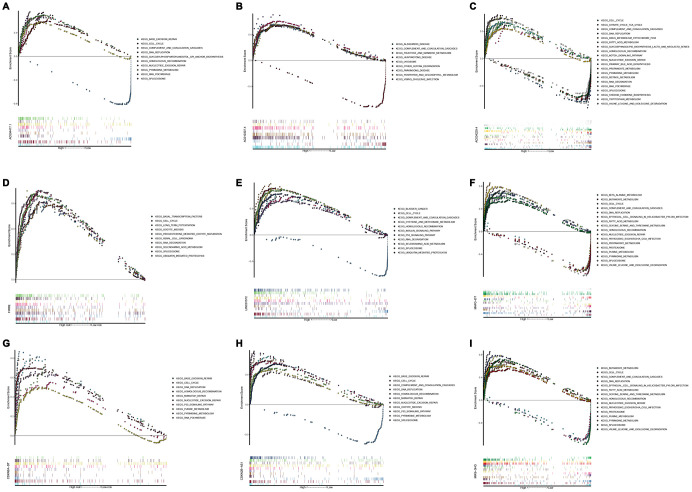
**Gene set enrichment analysis of lncRNAs of recurrence related classifier.** GSEA results showed in (**A**) AC004477.1, (**B**) AC010307.4, (**C**) AC034229.4, (**D**) FIRRE, (**E**) LINC01572, (**F**) MAFG−DT, (**G**) CDKN2A−DT, (**H**) CDKN2B−AS1, and (**I**) MIR9−3HG. GSEA, Gene set enrichment analysis.

In order to investigate different activated KEGG signaling pathways in HCC, GSEA was performed on 8 OS-related lncRNAs and 14 recurrence-related lncRNAs expression datasets. We considered the difference as statistically significant when | NES | ≥ 1, NOM p-value <0.01 and FDR q-val <0.25. All significant enrichment pathways were listed in Supplementary Tables 7 and 8. Figure 6 showed the most significant KEGG pathway function enrichment of 8-OS-related lncRNAs. Figure 7 showed the most significant KEGG pathway function enrichment of 14-recurrence-related lncRNAs. The results showed that Aminoacyl TRNA biosynthesis, Arginine and proline metabolism, Basal transcription factor, Base excision repair, Bladder cancer, Cytoplasmic DNA sensing pathway, DNA replication, Epithelial Signaling in Helicobacter Pylori Infection, Focal adhesion, Gap junction, homologous recombination, leukocyte transendothelial migration, Mapk signaling pathway, Nod like receptor signaling pathway, p53 signaling pathway, pathways in cancer, nucleotide excision repair, RNA degradation, cell cycle, spliceosome, VEGF signaling pathway, and WNT signaling pathways showed consistent enrichment in the up-regulated phenotypes of 8-OS-related lncRNAs (Figure 6). The results showed that Basal transcription factor, Base excision repair, DNA replication, mismatch repair, Lysosome, proteasome, homologous recombination, leukocyte transendothelial migration, P53 signaling pathway, pathways in cancer, Nucleotide excision repair, RNA degradation, cell cycle, and spliceosome signaling pathways showed consistent enrichment in the up-regulated phenotypes of 14-recurrence-related lncRNAs (Figure 7).

## DISCUSSION

HCC is a malignant tumor with high heterogeneity, which adds to the difficulty of prognosis and treatment [14]. The progression of hepatocellular carcinoma involves genetic and epigenetic changes, which are closely related to the poor prognosis of HCC [15]. Previous studies have shown that commonly used clinicopathological parameters (such as age, TNM staging, sex, viral infection, and AFP levels) were not sufficient to accurately predict prognosis of patients. Emerging evidence illustrates that lncRNA plays a critical role in regulating the progression of hepatocellular carcinoma (HCC) [16, 17]. Some studies have been conducted from the genetic perspective to screen lncRNA as a biomarker of HCC in the past few decades [18, 19]. To date, however, studies have attempted to predict the prognosis of patients by gene signature, but limited by sample size, biological heterogeneity, inappropriate data processing methods, and validation methods, there was usually not a good prediction ability.

In this study, we developed two lncRNA-based classifiers to predict survival and recurrence, respectively. Compared with previous studies, our research has the following advantages. First, we included 312 patients in the OS group and 269 patients in the recurrence group to reduce the deviation caused by insufficient sample size. Since only a small number of lncRNA were identified in previous studies, 15113 lncRNAs were isolated from the gene expression profile of LICH data set in this study. Furthermore, in order to identify useful lncRNA markers in high-dimensional data sets, an appropriate approach is required. The LASSO-Cox regression model was a popular tool for regression using high-dimensional predictors, which can more effectively perform dimensionality analysis on high-throughput sequencing data to construct more accurate gene signatures. The experience expansion of LASSO punishment can reduce the error discovery rate in the high-dimensional Cox regression model [20]. Finally, in addition to factors such as age, gender, TNM stage, and tumor stage, we also analyze AFP, HBV, HCV, Alcohol, Family history, Fibrosis Ishak score/Liver cirrhosis, BMI, Platelet result, Performance status, Child-Pugh grade, ALB, Region/Race, Adjacent tissue inflammation, etc. Among them, AFP, HCV, Alcohol, Family history, Fibrosis Ishak score/Liver cirrhosis, BMI, Platelet result, ALB, Region/Race, Adjacent tissue inflammation are not independent factors with significant statistical differences. The lack of data in some of these samples may affect the results, and larger sample studies are still needed. In conclusion, the prognostic ability of classifiers in this study is more reliable and accurate than previous studies. In addition, the nomogram risk score based on classifier and clinical characterization as a method to predict prognosis provides a visual method for predicting OS and recurrence in HCC patients. The nomogram based on 8-lncRNAs-based classifier combined with TNM M stage and performance status can visually predict OS, and a 14-lncRNAs-based classifier combined with TNM M stage and performance status can be used to visually predict recurrence, both having excellent predictive power and accuracy.

Some of the lncRNAs involved in this study have been investigated in past studies. AP002478.1 can predict hepatitis virus positive HCC as prognostic targets [21]. Research by Lou et al suggests that C10orf91 is one of five lncRNAs expression as competing endogenous RNAs in regulating hepatoma carcinoma [22]. LINC01116 was significantly associated with HCC patients' poor outcomes [23]. CDKN2B-AS1 has been reported promotes tumor growth and metastasis of HCC by targeting let-7c-5p/NAP1L1 axis [24]. FIRRE has been reported in the literature to activate the Wnt/β-catenin signaling pathway to promote the growth of diffuse large B lymphoma cells by regulating the nuclear translocation of β-catenin [25]. Chen et al. found that LINC01572 can distinguish between early and advanced lung squamous cell carcinoma [26]. MIR9-3HG was considered to be related to the survival time of Head and neck squamous cell carcinoma in the study by Hu et al. [27]. These lncRNAs have been studied in a variety of cancers, including HCC. In the classifier of our study, AC090921.1, AC096637.2, LINC01224, SERTAD4-AS1, AC004477.1, AC034229.4, AC209154.1, CDKN2A-DT, CDKN2B-AS1, LINC01549, MAFA-AS1, MAFA-AS1, MAFG-DT, MIR9-3HG and SNHG25 has not been reported to related to HCC biology, the functions and mechanisms of these lncRNAs in HCC need to be further investigated.

To further explore the function of the 20 lncRNAs in this study. GSEA was used to detect its genetic enrichment. KEGG pathway analysis showed that these genes are associated with rich metabolic pathways. Enrichment with phenotypic consistency was also found in pathways such as Aminoacyl TRNA biosynthesis, Arginine and proline metabolism, Basal transcription factor, Base excision repair, Bladder cancer, Cytoplasmic DNA sensing pathway, DNA replication, Epithelial Signaling in Helicobacter Pylori Infection, Focal adhesion, Gap junction, homologous recombination, leukocyte transendothelial migration, mapk signaling pathway, Nod like receptor signaling pathway, P53 signaling pathway, pathways in cancer, Nucleotide excision repair, RNA degradation, cell cycle, spliceosome, VEGF signaling pathway, and WNT signaling pathways. These results indicate that these 20 lncRNAs may participate in the occurrence and development of HCC through these pathways. As an important process of cell division and growth, the active DNA replication pathway promotes tumor growth and proliferation. Studies have shown that N7-alkyl-dG can block DNA replication, suggesting that these lncRNAs can be potential targets for tumor drug treatment [28]. In this study, multiple lncRNAs were enriched in the cell cycle pathway, and drugs acting on the cell cycle may benefit patients [29, 30]. However, further research is needed to investigate and verify the function of these 22 lncRNAs.

Current research inevitably has some limitations that can be explored in the future. First, we developed a lncRNAs-based classifier based on half of the LIHC data and used another part for verification, but the limited number of validation sets required a larger sample to further validate our model. Secondly, this study was based on a study of the TCGA database that determines a retrospective study, and a larger sample of more regional prospective studies was still needed. Third, in this study, the significance of lncRNAs in the development of HCC is unquestionable, but the mechanism behind it was not yet clear and further researches were needed. Moreover, the RNA sequencing data of this study were based on clinical specimens, which increase the difficulty of clinical application. Finally, whether it was TCGA's RNA-seq or GEO's Array chip, its expensive price was also an obstacle to clinical practice. If we could extract the lncRNA we need a more accessible blood sample, it would become a more potentially valuable method.

In conclusion, we proved that the lncRNA-based classifier devised by LASSO cox method can accurately predict survival and recurrence, and divide HCC patients into low- and high-risk groups. Furthermore, the novel nomogram constructed based on this classifier combined with clinical characterization can not only visually predict HCC survival and recurrence, but also increase its prognostic value, making it a potentially valuable biomarker signature in clinical practice.

## MATERIALS AND METHODS

### Patient data

Liver Hepatocellular Carcinoma (LIHC) read counts data was downloaded from TCGA, a publicly available portal (up to May 10, 2019, https://tcga-data.nci.nih.gov/tcga/). Forty-nine adjacent non-tumor samples and 368 HCC samples were obtained after the removal of non-HCC patients and patients who lost critical data. Clinical characteristics of patients were obtained from the cBioportal platform (http://cbioportal.org/) [31], A web resource for visual exploration and analysis multidimensional cancer genome data. The exclusion criteria were as follows: 1) not HCC samples; 2) samples with clinical data but without lncRNA sequence data; 3) samples missing important clinical or biological data; and 4) Patients were followed up for less than three months. After the removal of non-HCC patients and patients lacking critical information, 312 patients were finally reserved for further study. The expression matrices for GSE76427 and GSE116174 were downloaded from the Gene Expression Omnibus (GEO) database (https://www.ncbi.nlm.nih.gov/geo/). The expression matrices for GSE76427 and GSE116174 were downloaded from the Gene Expression Omnibus (GEO) database (https://www.ncbi.nlm.nih.gov/geo/). GSE116174 contains 64 patients with OS > 90 days. In GSE76427, 81 patients with recurrence follow-up longer than 90 days were used for further analysis.

### Data processing

LncRNAs of LIHC set was re-annotated by the gene annotation file " gencode.v30.long_noncoding_RNAs ", which is Downloaded from the gencode website. 15113 lncRNAs were identified from LIHC set. The expression value of each lncRNA was normalized with the TMM function of the ‘limma’ and ‘edgeR’ package for further analysis [32]. We used the ‘edgeR’ package to test all the data to identify lncRNAs that were differentially expressed by |logFC| >2 and padj < 0.05 in the tumor compared with normal samples. Then, the differentially expressed lncRNAs (DElncRNAs) were subjected to univariate Cox regression, and lncRNAs with p < 0.05 were identified as prognostic DElncRNAs for further research. GEO's lncRNAs were identified by the gpl platform annotation file and the Fasta file "gencode.v32.lncRNA_transcripts.fa".3494 lncRNAs were identified from GSE76427 and 5690 lncRNAs were identified from GSE116174. The OS-related prognostic DElncRNAs that intersect with GSE116174 are used for the development of OS-classifiers. Prognostic DElncRNAs associated with recurrences that intersect with GSE76427 are used to construct recurrence-classifiers. Because the detection methods used by the TCGA database and the GEO database are different, the background noise is also different. So we log2 (x + 1) the TCGA data set and normalize it using the zscore method. zscore normalization is also performed in the GEO dataset. The "sva" package is used to remove batch effects between TCGA and GEO datasets.

### Construction of lncRNAs classifier

LASSO is a commonly used high-dimensional predictive regression method. The method is a compression estimation. By constructing a penalty function, it can get a more refined model, and make it compress some regression coefficients, that is, the sum of the absolute values of the forcing coefficients is less than a certain fixed value. Also, set some regression coefficients to be zero. Therefore, it retains the advantage of subset contraction and is a biased estimation for processing data with complex collinearity [33]. The lncRNAs related to OS and recurrence were identified by LASSO COX regression model [34]. The regression coefficients (β) of each related lncRNAs are reserved for the development of lncRNAs-based classifier. The lncRNAs based classifier = ∑ EXP(lncRNA) * β. Based on the optimal cut-off value calculated by x-tile software version 3.6.1 (Yale University School of Medicine, New Haven, CT, USA), the LIHC set was divided into high and low risk groups [35]. The time-dependent receiver operating characteristic (tdROC) curve analysis, calibration curve analysis, Kaplan-Meier survival analysis were used to evaluate predictive ability of the models in training cohort, test cohort, TCGA cohort, and the GEO cohort. After that, we began to construct genomic-clinical nomograms to predict the prognosis and mortality of each HCC patient individually [36].

### Data analysis

The Chi-square test, COX survival analysis, and other data processing were completed by SPSS 19.0. Kaplan-Meier log rank test was calculated by medcalc (Version 19.0). Time-dependent ROC (tdROC) was used to assess the performance of lncRNA-based classifier with “time ROC” package in R software(Version 3.6.1). And area under ROC (AUC) was used to assess the accuracy of the prediction. Calibration curve and C-index are performed by ‘rms’ package. The larger C-index indicates that the prediction model has better accuracy [37]. ‘Hmisc’, ‘rms’, and ‘survival’ were used to develop a nomogram. When all the hypotheses are P < 0.05, the difference is statistically significant.

### Gene set enrichment analysis

To identify the activation of different KEGG signaling pathways in HCC, we conducted GSEA between down-regulated and up-regulated phenotypes. The lncRNAs of the classifier were divided into up- and down-regulated groups by median. Gene Set Enrichment Analysis (GSEA) was performed by JAVA program GSEA 4.0.2 with the MSigDB Collection (c2.cp.kegg.v7.0.symbol). Normalized enrichment score (NES), nominal p-value and false discovery rate (FDR) were used to quantify enrichment magnitude and statistical significance, respectively [38]. When | NES | ≥ 1, FDR q-val <0.25 and NOM p-value <0.01 were considered significant.

## Supplementary Material

Supplementary Figures

Supplementary Table 1

Supplementary Tables 2-6, 9

Supplementary Table 7

Supplementary Table 8

Supplementary Table 10

Supplementary Table 11

Supplementary Table 12
