# Supplementary material for: A novel molecular-clinicopathologic nomogram to improve prognosis prediction of hepatocellular carcinoma
Source: Aging (Albany NY). 2020 Jun 30;12(13):12896–920. doi: 10.18632/aging.103350 (PMC7377850; doi:10.18632/aging.103350)
Supplement: Supplementary Figures [file aging-12-103350-s008..pdf]

SUPPLEMENTARY FIGURES

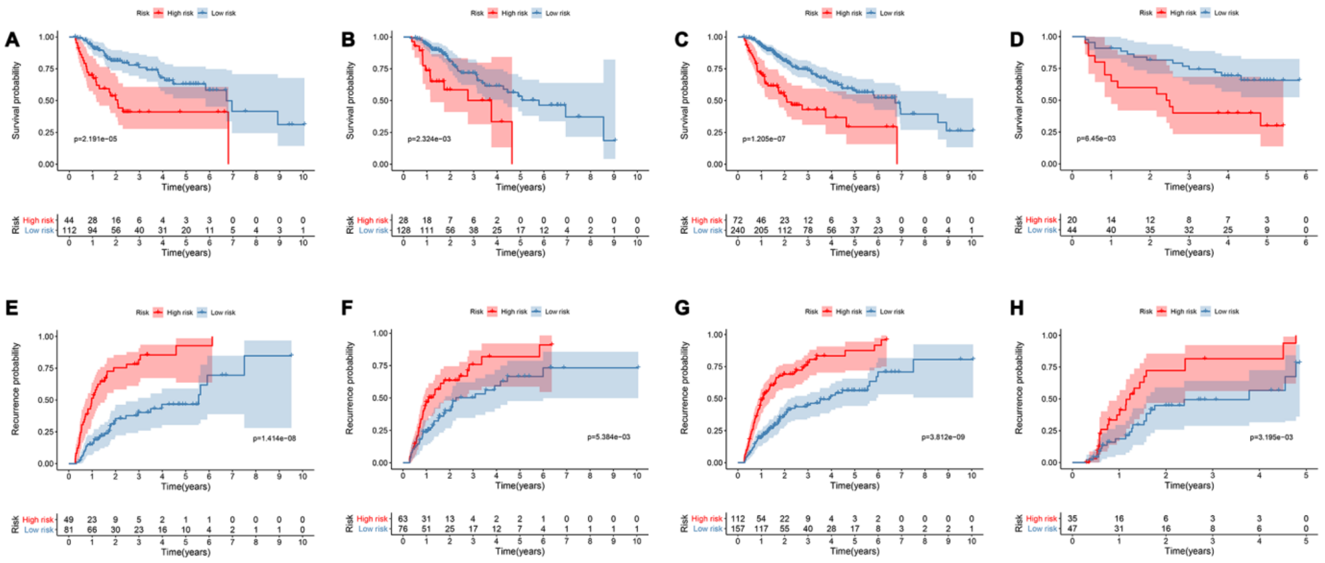

Supplementary Figure 1. Kaplan-Meier analysis in the training, validation and whole cohorts according to the lncRNA-based classifiers. Kaplan-meier survival analysis was performed to predict overall survival in the (**A**) training cohort, (**B**) test cohort, (**C**) TCGA cohort and (**D**) GEO cohort, according to the high-risk and low-risk groups stratified by the 8-lncRNAs-based classifier. Kaplan-meier survival analysis was performed to predict recurrence in the (**E**) training cohort, (**F**) test cohort, (**G**) TCGA cohort and (**H**) GEO cohort,, according to the high-risk and low-risk groups stratified by the 14-lncRNAs-based classifier.

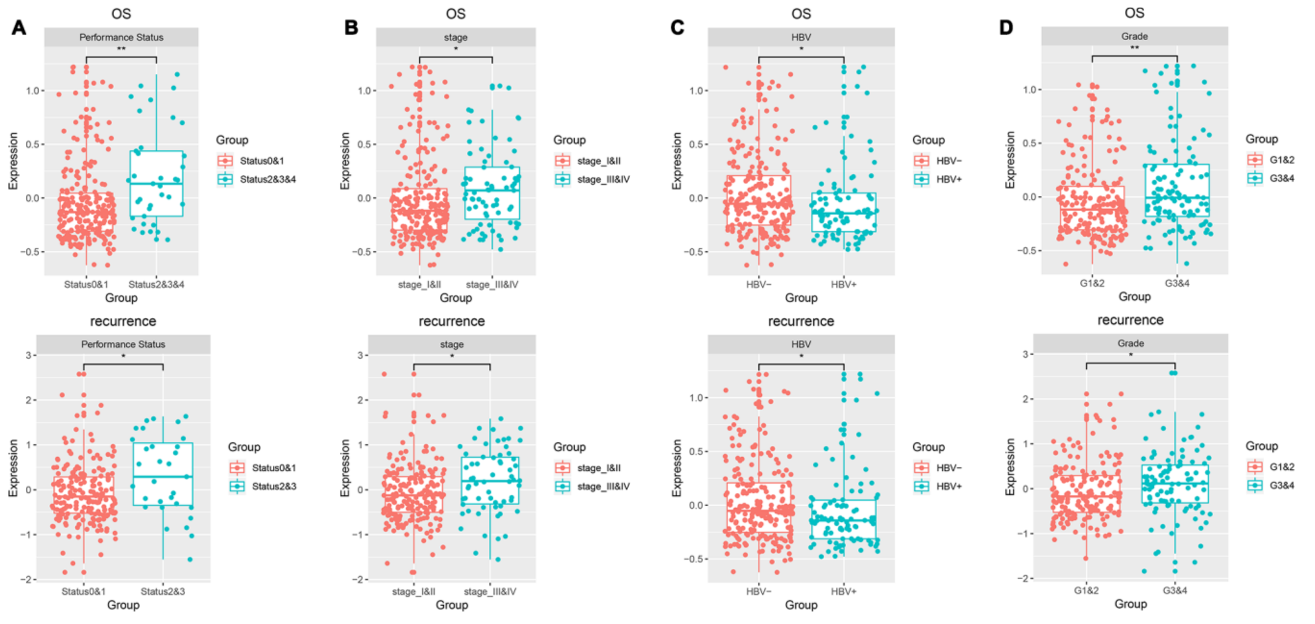

Supplementary Figure 2. Boxplot of lncRNA-based classifier score in patients with clinicopathological risk factors. Boxplot of 8-lncRNAs-based classifier score and 14-lncRNAs-based classifier score in patients with (**A**) Performance Status, (**B**) TNM stage, (**C**) HBV, and (**D**) grade.

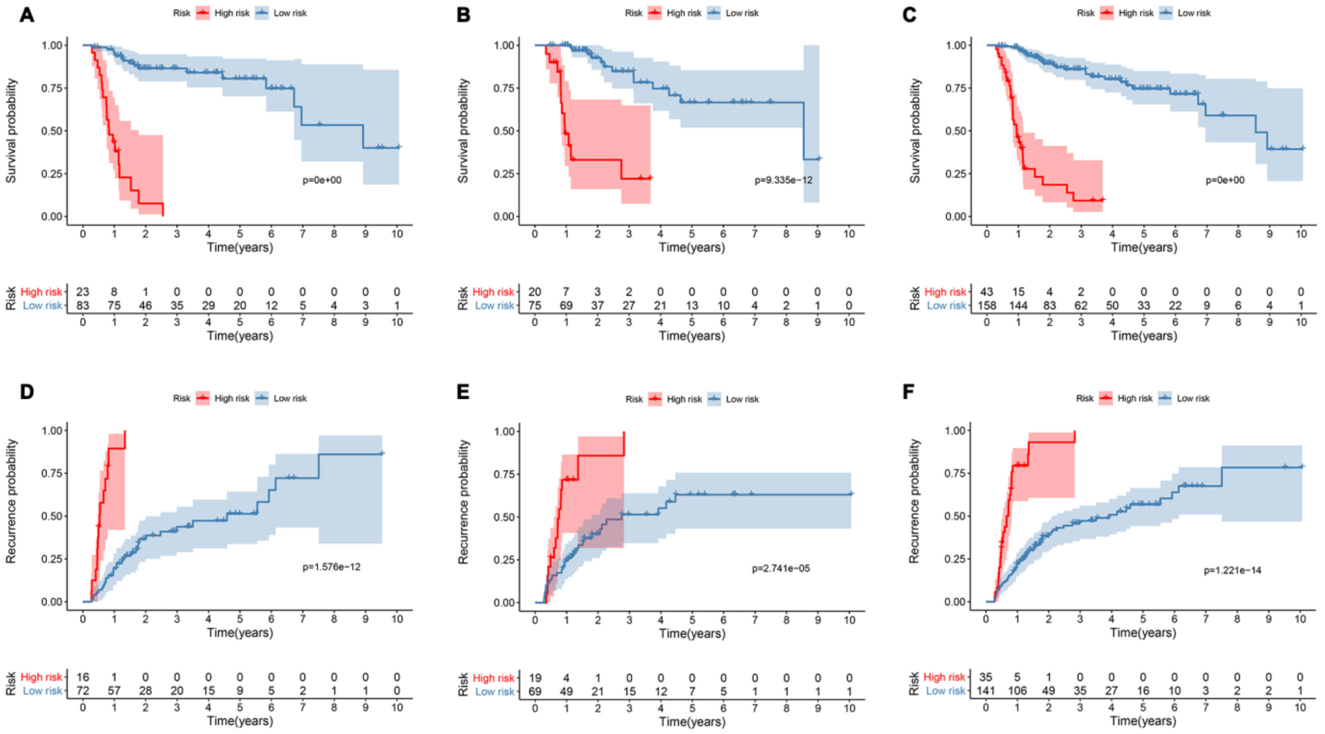

Supplementary Figure 3. Kaplan-Meier analysis in the training, validation and whole cohorts according to the molecular-clinicopathological nomograms. Kaplan-meier survival analysis was performed to predict overall survival in the (**A**) training cohort, (**B**) test cohort, and (**C**) TCGA cohort, according to the high-risk and low-risk groups stratified by the OS-nomogram. Kaplan-meier survival analysis was performed to predict recurrence in the (**D**) training cohort, (**E**) test cohort, and (**F**) TCGA cohort, according to the high-risk and low-risk groups stratified by the recurrence-nomogram. OS, overall survival.

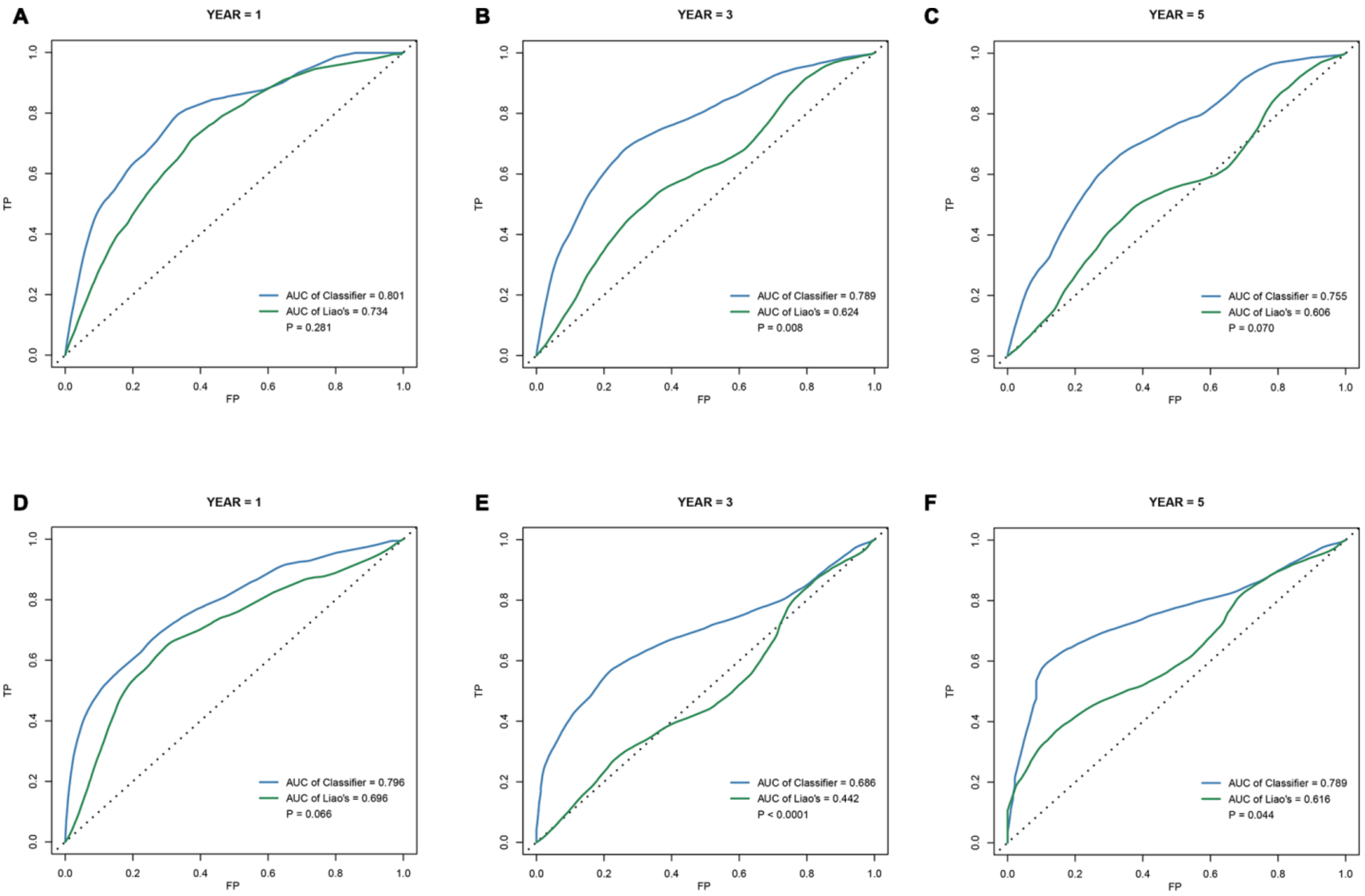

Supplementary Figure 4. Comparison of Classifiers and Liao's Biomarkers. (**A**–**C**) The 1, 3, and 5-year Time-dependent ROC curves compare the prognostic accuracy of the OS-related Classifier and Liao's Biomarkers (**D**–**F**) The 1, 3, and 5-year Time-dependent ROC curves compare the prognostic accuracy of the recurrence -related Classifier and Liao's Biomarkers; OS, overall survival; lncRNA, long non-coding RNA; ROC, receiver operating characteristic.
