# Supplementary material for: A novel molecular-clinicopathologic nomogram to improve prognosis prediction of hepatocellular carcinoma
Source: Aging (Albany NY). 2020 Jun 30;12(13):12896–920. doi: 10.18632/aging.103350 (PMC7377850; doi:10.18632/aging.103350)
Supplement: Supplementary Table 1 [file aging-12-103350-s005..docx]

**Supplementary Table 1.** **lncRNA differentially expressed in tumor vs. normal**

| **Gene** | **logFC** | **PValue** | **FDR** | **regulated** |
| --- | --- | --- | --- | --- |
| AC008080.1 | 2.337853227 | 1.26E-18 | 1.88E-14 | Up-Regulated |
| AC138356.1 | 2.409692286 | 2.60E-35 | 3.92E-31 | Up-Regulated |
| B3GALT5-AS1 | 2.340039337 | 1.36E-06 | 0.018360128 | Up-Regulated |
| AL357033.2 | 2.147950722 | 1.19E-06 | 0.016110541 | Up-Regulated |
| AC104809.1 | 2.544803963 | 2.06E-10 | 2.96E-06 | Up-Regulated |
| WARS2-IT1 | 2.010970604 | 3.29E-13 | 4.83E-09 | Up-Regulated |
| HTR2A-AS1 | 2.382702661 | 4.23E-16 | 6.29E-12 | Up-Regulated |
| AL358216.1 | 2.119632744 | 6.46E-08 | 0.000899747 | Up-Regulated |
| AC004540.2 | 2.449142953 | 4.98E-17 | 7.41E-13 | Up-Regulated |
| AL392089.1 | 2.004418132 | 3.84E-11 | 5.56E-07 | Up-Regulated |
| AC092155.1 | 3.012514559 | 4.36E-16 | 6.47E-12 | Up-Regulated |
| PCDH9-AS2 | 3.250985356 | 5.94E-09 | 8.41E-05 | Up-Regulated |
| CCDC26 | 2.106222161 | 3.53E-08 | 0.000494035 | Up-Regulated |
| AC016999.1 | 2.95263034 | 1.04E-31 | 1.56E-27 | Up-Regulated |
| AC004160.1 | 2.495710118 | 5.49E-20 | 8.23E-16 | Up-Regulated |
| AC004160.2 | 2.208930678 | 1.80E-09 | 2.56E-05 | Up-Regulated |
| LINC01818 | 2.693362723 | 1.29E-15 | 1.92E-11 | Up-Regulated |
| AC104024.1 | 2.090465686 | 1.56E-10 | 2.25E-06 | Up-Regulated |
| HAO2-IT1 | 2.337257553 | 3.57E-13 | 5.24E-09 | Up-Regulated |
| NCOA7-AS1 | 2.366738788 | 1.67E-11 | 2.42E-07 | Up-Regulated |
| AF165147.1 | 2.584552947 | 3.12E-35 | 4.71E-31 | Up-Regulated |
| AC003988.1 | 2.313057146 | 3.93E-07 | 0.005397929 | Up-Regulated |
| AL603840.1 | 2.52847096 | 1.16E-13 | 1.70E-09 | Up-Regulated |
| LINC01920 | 3.371026202 | 1.52E-10 | 2.19E-06 | Up-Regulated |
| AL121892.1 | 3.261998047 | 1.40E-14 | 2.07E-10 | Up-Regulated |
| HAND2-AS1 | 2.393040984 | 8.44E-15 | 1.25E-10 | Up-Regulated |
| AL157832.2 | 2.096589985 | 9.18E-13 | 1.34E-08 | Up-Regulated |
| TMEM26-AS1 | 2.020833447 | 2.12E-15 | 3.14E-11 | Up-Regulated |
| AC006960.2 | 4.193422758 | 9.52E-13 | 1.40E-08 | Up-Regulated |
| ST6GAL2-IT1 | 2.399081071 | 2.85E-06 | 0.038210365 | Up-Regulated |
| CLRN1-AS1 | 2.081021895 | 1.06E-08 | 0.00014879 | Up-Regulated |
| LINC02027 | 2.320188417 | 4.02E-10 | 5.76E-06 | Up-Regulated |
| AC079061.1 | 2.289446661 | 9.97E-17 | 1.48E-12 | Up-Regulated |
| LINC02275 | 2.235005424 | 3.54E-11 | 5.12E-07 | Up-Regulated |
| AC010280.1 | 2.277340475 | 3.77E-11 | 5.46E-07 | Up-Regulated |
| LINC02428 | 2.120522753 | 9.21E-16 | 1.37E-11 | Up-Regulated |
| AC010280.2 | 2.723110072 | 2.66E-26 | 4.01E-22 | Up-Regulated |
| AC097537.1 | 3.031803213 | 1.40E-07 | 0.001933659 | Up-Regulated |
| LINC01093 | 3.605149756 | 2.12E-40 | 3.21E-36 | Up-Regulated |
| AC105384.1 | 2.147788376 | 5.67E-12 | 8.27E-08 | Up-Regulated |
| LINC01612 | 3.145066748 | 3.83E-09 | 5.43E-05 | Up-Regulated |
| AC093725.2 | 3.866445155 | 1.56E-11 | 2.27E-07 | Up-Regulated |
| AC107396.1 | 4.434859488 | 8.55E-15 | 1.26E-10 | Up-Regulated |
| AC015468.1 | 3.090611562 | 2.94E-11 | 4.26E-07 | Up-Regulated |
| LINC02153 | 2.615956555 | 4.85E-07 | 0.00663917 | Up-Regulated |
| AC245519.1 | 2.794982599 | 9.30E-07 | 0.012662771 | Up-Regulated |
| KBTBD11-OT1 | 2.215220985 | 1.67E-11 | 2.43E-07 | Up-Regulated |
| AC245187.1 | 3.026122532 | 3.72E-08 | 0.000520309 | Up-Regulated |
| LINC01863 | 2.479909577 | 1.98E-12 | 2.89E-08 | Up-Regulated |
| AC015468.4 | 3.015076703 | 1.54E-09 | 2.20E-05 | Up-Regulated |
| AP001257.1 | 2.017647616 | 6.26E-07 | 0.008551925 | Up-Regulated |
| AC026369.3 | 2.687602085 | 4.47E-22 | 6.71E-18 | Up-Regulated |
| AL161668.4 | 2.056100138 | 9.41E-23 | 1.41E-18 | Up-Regulated |
| AC092384.2 | 2.449851946 | 1.56E-15 | 2.31E-11 | Up-Regulated |
| AC023158.1 | 3.065051821 | 8.16E-10 | 1.17E-05 | Up-Regulated |
| AC099508.2 | 2.556858217 | 4.60E-19 | 6.88E-15 | Up-Regulated |
| AC009063.3 | 2.633545827 | 7.36E-09 | 0.000103953 | Up-Regulated |
| AC087392.1 | 2.576898746 | 4.47E-17 | 6.66E-13 | Up-Regulated |
| GTSCR1 | 2.097092028 | 3.71E-07 | 0.005093861 | Up-Regulated |
| LINC00683 | 2.15608051 | 1.52E-08 | 0.000213688 | Up-Regulated |
| LINC00907 | 2.966675857 | 3.98E-30 | 6.00E-26 | Up-Regulated |
| AC090227.2 | 2.694737524 | 5.30E-12 | 7.73E-08 | Up-Regulated |
| AC010776.2 | 3.702580026 | 9.88E-16 | 1.47E-11 | Up-Regulated |
| FENDRR | 2.794694543 | 5.32E-26 | 8.01E-22 | Up-Regulated |
| AC135012.3 | 2.616525124 | 3.51E-06 | 0.047013894 | Up-Regulated |
| AC245128.3 | 2.109099852 | 7.59E-14 | 1.12E-09 | Up-Regulated |
| AL021328.1 | 2.263295653 | 2.01E-09 | 2.86E-05 | Up-Regulated |
| AC010776.3 | 3.323126706 | 7.80E-09 | 0.00011021 | Up-Regulated |
| AC008556.1 | 2.1407398 | 6.92E-46 | 1.05E-41 | Up-Regulated |
| AC245123.1 | 2.628736817 | 4.88E-09 | 6.92E-05 | Up-Regulated |
| KLHL30-AS1 | 7.339018569 | 5.37E-71 | 8.11E-67 | Up-Regulated |
| AL358613.2 | 2.241143364 | 3.17E-10 | 4.56E-06 | Up-Regulated |
| AC080100.1 | 2.016001718 | 1.39E-09 | 1.99E-05 | Up-Regulated |
| AP004782.1 | 3.291381357 | 1.10E-22 | 1.66E-18 | Up-Regulated |
| PART1 | -7.090922404 | 2.56E-09 | 3.65E-05 | Down-Regulated |
| LINC01116 | -3.208507258 | 1.41E-15 | 2.09E-11 | Down-Regulated |
| IGF2BP2-AS1 | -3.335393794 | 2.89E-09 | 4.11E-05 | Down-Regulated |
| CCDC13-AS1 | -2.126504172 | 3.68E-08 | 0.00051462 | Down-Regulated |
| FAM182B | -2.008802319 | 3.63E-11 | 5.26E-07 | Down-Regulated |
| AC011944.1 | -2.520197238 | 9.11E-07 | 0.012397329 | Down-Regulated |
| C10orf91 | -4.494080427 | 3.04E-07 | 0.004187145 | Down-Regulated |
| FAM87A | -3.102318567 | 9.43E-09 | 0.000132987 | Down-Regulated |
| TSPEAR-AS2 | -2.578579944 | 8.57E-13 | 1.26E-08 | Down-Regulated |
| DSCR4 | -7.740209142 | 1.63E-06 | 0.021965852 | Down-Regulated |
| LINC00308 | -4.54720971 | 7.32E-09 | 0.000103398 | Down-Regulated |
| C17orf82 | -2.837083167 | 1.19E-29 | 1.79E-25 | Down-Regulated |
| TCL6 | -2.654784206 | 2.30E-07 | 0.003169763 | Down-Regulated |
| AC091057.1 | -2.038721874 | 3.51E-23 | 5.27E-19 | Down-Regulated |
| AC069277.1 | -5.230803103 | 4.84E-09 | 6.86E-05 | Down-Regulated |
| LINC00173 | -2.014664824 | 5.93E-10 | 8.49E-06 | Down-Regulated |
| NPSR1-AS1 | -6.460808421 | 2.37E-17 | 3.54E-13 | Down-Regulated |
| DSCR8 | -7.759397426 | 1.21E-11 | 1.76E-07 | Down-Regulated |
| AL590705.1 | -2.303188633 | 2.99E-11 | 4.33E-07 | Down-Regulated |
| LINC00501 | -2.902063761 | 1.82E-06 | 0.02449293 | Down-Regulated |
| IBA57-DT | -2.083657809 | 3.99E-17 | 5.94E-13 | Down-Regulated |
| SERTAD4-AS1 | -2.83729754 | 3.12E-13 | 4.58E-09 | Down-Regulated |
| SLC12A5-AS1 | -2.303314065 | 8.83E-11 | 1.27E-06 | Down-Regulated |
| TDRG1 | -6.100619231 | 2.04E-08 | 0.000286452 | Down-Regulated |
| ST8SIA6-AS1 | -5.571901613 | 7.92E-21 | 1.19E-16 | Down-Regulated |
| LINC01121 | -4.339705648 | 1.07E-14 | 1.58E-10 | Down-Regulated |
| LINC01446 | -4.515523642 | 4.42E-08 | 0.000616657 | Down-Regulated |
| AL359878.1 | -2.283539057 | 6.51E-18 | 9.72E-14 | Down-Regulated |
| DUXAP8 | -3.006718901 | 7.60E-19 | 1.14E-14 | Down-Regulated |
| Z97192.2 | -2.360041084 | 8.17E-08 | 0.00113669 | Down-Regulated |
| FIRRE | -3.130720827 | 3.10E-14 | 4.58E-10 | Down-Regulated |
| AC007277.1 | -3.802346908 | 3.66E-11 | 5.29E-07 | Down-Regulated |
| LINC00887 | -2.015906569 | 5.60E-07 | 0.007659894 | Down-Regulated |
| LINC00488 | -3.514328249 | 1.02E-06 | 0.013844273 | Down-Regulated |
| AC090921.1 | -4.675744778 | 1.28E-07 | 0.001772606 | Down-Regulated |
| LINC01139 | -3.637445414 | 1.85E-06 | 0.024924934 | Down-Regulated |
| AC073263.1 | -2.210462589 | 1.53E-08 | 0.00021505 | Down-Regulated |
| AC099552.1 | -7.99121428 | 1.22E-07 | 0.001689884 | Down-Regulated |
| AC008060.1 | -6.168222177 | 1.88E-06 | 0.025324187 | Down-Regulated |
| LINC00397 | -3.460537417 | 3.65E-06 | 0.048805071 | Down-Regulated |
| LINC01615 | -2.926565985 | 2.96E-09 | 4.20E-05 | Down-Regulated |
| AC005165.1 | -3.517152985 | 4.45E-08 | 0.000621051 | Down-Regulated |
| DSCR4-IT1 | -6.373724714 | 1.18E-06 | 0.015938637 | Down-Regulated |
| AL513217.1 | -2.484689634 | 1.11E-08 | 0.000156104 | Down-Regulated |
| LINC00114 | -2.929844217 | 3.46E-07 | 0.004753226 | Down-Regulated |
| AC004870.2 | -7.229326119 | 7.52E-07 | 0.010262349 | Down-Regulated |
| AC147651.1 | -2.828200581 | 1.15E-16 | 1.71E-12 | Down-Regulated |
| LINC02561 | -3.353435667 | 2.92E-17 | 4.36E-13 | Down-Regulated |
| MIR548XHG | -7.272816951 | 1.29E-08 | 0.000181263 | Down-Regulated |
| HAGLR | -6.143084038 | 8.22E-30 | 1.24E-25 | Down-Regulated |
| AL117329.1 | -5.789454869 | 2.89E-11 | 4.19E-07 | Down-Regulated |
| AC114489.1 | -5.719125093 | 1.87E-08 | 0.000262753 | Down-Regulated |
| SLC25A34-AS1 | -2.32592584 | 2.94E-09 | 4.17E-05 | Down-Regulated |
| AC245100.1 | -2.373679521 | 2.33E-08 | 0.000326333 | Down-Regulated |
| LINC01117 | -2.944959684 | 2.62E-08 | 0.000367607 | Down-Regulated |
| LINC00853 | -2.194202 | 2.42E-19 | 3.63E-15 | Down-Regulated |
