# Supplementary material for: A novel molecular-clinicopathologic nomogram to improve prognosis prediction of hepatocellular carcinoma
Source: Aging (Albany NY). 2020 Jun 30;12(13):12896–920. doi: 10.18632/aging.103350 (PMC7377850; doi:10.18632/aging.103350)
Supplement: Supplementary Tables 2-6, 9 [file aging-12-103350-s007..pdf]

SUPPLEMENTARY TABLES

Supplementary Table 2. The detailed information of lncRNAs for constructing the prognostic signature.

Gene name	ENSG_ ID	Gene_type	bp	Chromosome	β	Cutoff Value
**8-lncRNA-based classifier for OS**						**0.2**
AC090921.1	ENSG00000214803	lincRNA	1195	Chromosome 8: 124,192,671-124,247,398	0.0299	0.7
AC096637.2	ENSG00000265415	antisense	1846	Chromosome 17: 59,202,677-59,203,829	0.0125	0.8
AP002478.1	ENSG00000266401	antisense	1455	Chromosome 18: 3,653,030-3,656,282	0.1838	1.5
C10orf91	ENSG00000180066	lincRNA	1846	Chromosome10: 132,444,327-132,449,408	0.2221	1.3
LINC01116	ENSG00000163364	lincRNA	1502	Chromosome 21: 44,477,850-44,478,493	0.0437	1.4
LINC01224	ENSG00000269416	lincRNA	2766	Chromosome 19: 23,399,233-23,416,075	0.0251	1.2
MAFG-DT	ENSG00000265688	bidirectional_promoter_ lncRNA	1895	Chromosome 17: 81,927,829-81,930,753	0.0137	1.3
SERTAD4-AS1	ENSG00000203706	antisense	726	Chromosome 1: 210,231,456-210,234,047	-0.1168	-0.9
**14-lncRNA-based classifier for recurrence**						**0.1**
AC004477.1	ENSG00000263412	processed_transcript	2910	Chromosome 17: 48,045,141-48,048,073	-0.0255	0.6
AC010307.4	ENSG00000250244	antisense	389	Chromosome 5: 133,256,492-133,275,977	0.1647	1.5
AC034229.4	ENSG00000272417	lincRNA	441	Chromosome 5: 10,203,600-10,204,040	0.0416	1.0
AC209154.1	ENSG00000276399	lincRNA	3801	Chromosome 17: 22,406,019-22,413,744	0.1580	0.3
C10orf91	ENSG00000180066	lincRNA	1846	Chromosome10: 132,444,327-132,449,408	0.3958	0.5
CDKN2A-DT	ENSG00000224854	antisense	823	Chromosome 9: 21,966,929-21,967,751	0.0233	0.7
CDKN2B-AS1	ENSG00000240498	antisense	7173	Chromosome 9: 21,994,139-22,128,103	0.0037	0.7
FIRRE	ENSG00000213468	processed_transcript	5506	Chromosome X: 131,688,779-131,830,862	0.00057	1.1
LINC01549	ENSG00000232560	lincRNA	1702	Chromosome 21: 17,438,821-17,450,104	-0.1140	-1.1
LINC01572	ENSG00000261008	lincRNA	3298	Chromosome 16: 72,236,281-72,665,014	0.1813	0.5
MAFA-AS1	ENSG00000254338	antisense	417	Chromosome 8: 143,417,679-143,419,150	0.0958	1.3
MAFG-DT	ENSG00000265688	bidirectional_promoter_ln cRNA	1895	Chromosome 17: 81,927,829-81,930,753	0.1348	-0.8
MIR9-3HG	ENSG00000255571	lincRNA	5607	Chromosome 15: 89,361,579-89,398,487	-0.365	-0.7
SNHG25	ENSG00000266402	lincRNA	278	Chromosome 17: 64,145,970-64,146,476	-0.0761	0.4
LINC02499	ENSG00000250436.1	lincRNA	763	Chromosome 4: 73,508,803-73,534,128	-0.19279	

Supplementary Table 3. Correlation points about nomogram prediction of overall survival.

Performance Status	Points	riskScore	Points	Total Points	1-year Survival Probability	Total Points	3-year Survival Probability	Total Points	5-year Survival Probability
0	64	-0.8	100	70	0.1	105	0.1	122	0.1
1	48	-0.6	91	84	0.2	119	0.2	135	0.2
2	32	-0.4	82	93	0.3	128	0.3	145	0.3
3	16	-0.2	73	102	0.4	137	0.4	153	0.4
4	0	0	64	110	0.5	145	0.5	161	0.5
**M**	**Points**	0.2	55	117	0.6	152	0.6	169	0.6
0	47	0.4	45	126	0.7	161	0.7	177	0.7
1	0	0.6	36	135	0.8	170	0.8	187	0.8
		0.8	27	149	0.9	184	0.9	200	0.9
		1	18						
		1.2	9						
		1.4	0						

Supplementary Table 4. Correlation points of nomogram prediction of recurrence.

Performance Status	Points	riskScore	Points	Total Points	1-year Survival Probability	Total Points	3-year Survival Probability	Total Points	5-year Survival Probability
0	52	-2	100	55	0.1	99	0.1	103	0.05
1	34	-1.5	90	75	0.2	118	0.2	119	0.1
2	17	-1	80	89	0.3	132	0.3	130	0.15
3	0	-0.5	70	101	0.4	144	0.4	139	0.2
**M**	**Points**	0	60	112	0.5	155	0.5	146	0.25
0	43	0.5	50	123	0.6	167	0.6	153	0.3
1	0	1	40	135	0.7	178	0.7	159	0.35
		1.5	30	149	0.8			165	0.4
		2	20	168	0.9			170	0.45
		2.5	10					176	0.5
		3	0					181	0.55
								187	0.6

Supplementary Table 5. Log rank test of 8-lncRNA-based classifier combined with performance status

Log Rank (Mantel-Cox)	group	low+Status0&1		low+Status2&3&4		high+Status0&1		high+Status2&3&4	
		chi-square	P values	chi-square	P values	chi-square	P values	chi-square	P values
Training cohort	low+Status0&1			11.3	0.001	15.511	0	34.829	0
	low+Status2&3&4	11.3	0.001			0.003	0.959	4.066	0.044
	high+Status0&1	15.511	0	0.003	0.959			4.208	0.04
	high+Status2&3&4	34.829	0	4.066	0.044	4.208	0.04		
Test cohort	low+Status0&1			19.896	0	2.102	0.147	65.568	0
	low+Status2&3&4	19.896	0			2.489	0.115	1.502	0.22
	high+Status0&1	2.102	0.147	2.489	0.115			12.862	0
	high+Status2&3&4	65.568	0	1.502	0.22	12.862	0		
TCGA cohort	low+Status0&1			30.199	0	18.938	0	108.295	0
	low+Status2&3&4	30.199	0			0.858	0.354	5.167	0.023
	high+Status0&1	18.938	0	0.858	0.354			14.022	0
	high+Status2&3&4	108.295	0	5.167	0.023	14.022	0		

Supplementary Table 6. Log rank test of 14-lncRNA-based classifier combined with performance status.

Log Rank (Mantel-Cox)	group	low+Status0&1		low+Status2&3		high+Status0&1		high+Status2&3	
		chi-square	P values	chi-square	P values	chi-square	P values	chi-square	P values
**Training cohort**	low+Status0&1			1.934	0.164	16.623	0	42.66	0
	low+Status2&3	1.934	0.164			0.079	0.778	2.609	0.106
	high+Status0&1	16.623	0	0.079	0.778			7.997	0.005
	high+Status2&3	42.66	0	2.609	0.106	7.997	0.005		
**Test cohort**	low+Status0&1			15.487	0	8.36	0.004	27.763	0
	low+Status2&3	15.487	0			2.423	0.12	0.334	0.563
	high+Status0&1	8.36	0.004	2.423	0.12			5.571	0.018
	high+Status2&3	27.763	0	0.334	0.563	5.571	0.018		
**TCGA cohort**	low+Status0&1			19.964	0	24.73	0	65.312	0
	low+Status2&3	19.964	0			1.395	0.238	3.5	0.061
	high+Status0&1	24.73	0	1.395	0.238			14.741	0
	high+Status2&3	65.312	0	3.5	0.061	14.741	0		

Supplementary Table 9. Correlation between overall survival-classifier-related lncRNAs and recurrence-classifier-related lncRNAs.

lncRNA-based classifier for OS	lncRNA-based classifier for recurrence	cor	p value
AC090921.1	AC034229.4	0.208874156	0.000578559
AC090921.1	AC209154.1	0.134326063	0.027898715
AC090921.1	C10orf91	0.21696736	0.000346205
AC090921.1	LINC01549	-0.167598529	0.005952943
AC090921.1	MAFA-AS1	0.145372058	0.017247764
AC090921.1	MAFG-DT	0.180382687	0.003041263
AC096637.2	AC034229.4	0.285522048	2.02E-06
AC096637.2	C10orf91	0.230304099	0.000142449
AC096637.2	CDKN2A-DT	0.143301482	0.018919805
AC096637.2	LINC01572	0.12455441	0.041604634
AC096637.2	MAFA-AS1	0.131372909	0.031559886
AC096637.2	MAFG-DT	0.28949342	1.43E-06
AC096637.2	SNHG25	0.198976	0.001056771
AP002478.1	AC004477.1	0.259711142	1.66E-05
AP002478.1	AC010307.4	0.259539077	1.69E-05
AP002478.1	AC209154.1	0.244094535	5.38E-05
AP002478.1	C10orf91	0.140072512	0.021808497
AP002478.1	CDKN2A-DT	0.172834139	0.004545744
AP002478.1	CDKN2B-AS1	0.163611629	0.007274098
AP002478.1	FIRRE	0.146847125	0.016136627
AP002478.1	LINC01572	0.238742496	7.90E-05
AP002478.1	MAFG-DT	0.293178891	1.03E-06
C10orf91	AC004477.1	0.169090332	0.005516772
C10orf91	AC010307.4	0.223319151	0.000228295
C10orf91	AC034229.4	0.224312505	0.000213672
C10orf91	CDKN2B-AS1	0.130183888	0.033145673
C10orf91	FIRRE	0.170799934	0.005052355
C10orf91	LINC01549	-0.218043958	0.000322882
C10orf91	LINC01572	0.149601672	0.014228252
C10orf91	MAFA-AS1	0.169959418	0.005276101
C10orf91	MAFG-DT	0.241248479	6.61E-05
C10orf91	MIR9-3HG	0.189558585	0.001826946
LINC01116	C10orf91	0.271001376	6.79E-06
LINC01116	MAFG-DT	0.131794777	0.031013017
LINC01224	AC034229.4	0.29166297	1.18E-06
LINC01224	AC209154.1	0.168876724	0.005577414
LINC01224	C10orf91	0.241786227	6.36E-05
LINC01224	FIRRE	0.243081896	5.79E-05
LINC01224	LINC01572	0.149374266	0.014377938
LINC01224	MAFA-AS1	0.270744672	6.93E-06
LINC01224	MAFG-DT	0.158255714	0.009458505
LINC01224	MIR9-3HG	0.257762954	1.93E-05
LINC01224	SNHG25	0.145502969	0.017146522
MAFG-DT	AC004477.1	0.217947287	0.000324915
MAFG-DT	AC010307.4	0.249828932	3.53E-05
MAFG-DT	AC034229.4	0.235960325	9.62E-05

MAFG-DT	C10orf91	0.241248479	6.61E-05
MAFG-DT	CDKN2A-DT	0.149773026	0.014116366
MAFG-DT	CDKN2B-AS1	0.120368864	0.049013373
MAFG-DT	LINC01572	0.197606609	0.001146105
MAFG-DT	MAFA-AS1	0.170178478	0.005216945
MAFG-DT	MIR9-3HG	0.19080438	0.001701746
MAFG-DT	SNHG25	0.23188722	0.000127749
SERTAD4-AS1	AC034229.4	-0.156188541	0.010446167
SERTAD4-AS1	C10orf91	-0.145826459	0.016898567
SERTAD4-AS1	CDKN2B-AS1	-0.133468183	0.02892273
SERTAD4-AS1	LINC01549	0.259481918	1.69E-05
SERTAD4-AS1	MAFA-AS1	-0.268111694	8.57E-06
SERTAD4-AS1	SNHG25	-0.140105715	0.021776952

Please browse Full Text version to see the data of Supplementary Tables 10–12.

Supplementary Table 10. Correlations between risk score of the 8-lncRNA-based classifier with overall survival and clinicopathological characteristics in training cohort, test cohort, TCGA cohort and GEO cohort.

Supplementary Table 11. Correlations between risk score of the 14-lncRNA-based classifier with recurrence and clinicopathological characteristics in training cohort, test cohort, TCGA cohort and GEO cohort.

Supplementary Table 12. Univariate and multivariate COX analyses of the lncRNA-based classifier for OS.
