# Supplementary material for: A novel molecular-clinicopathologic nomogram to improve prognosis prediction of hepatocellular carcinoma
Source: Aging (Albany NY). 2020 Jun 30;12(13):12896–920. doi: 10.18632/aging.103350 (PMC7377850; doi:10.18632/aging.103350)
Supplement: Supplementary Table 7 [file aging-12-103350-s001..docx]

**Supplementary Table 7.Gene sets enriched in overall survival related classifier**

| **Gene** | **Phenotype** | **Gene Set** | **NES** | **NOM p-val** | **FDR q-val** |
| --- | --- | --- | --- | --- | --- |
| AC090921.1 | up-regulated | KEGG_PATHOGENIC_ESCHERICHIA_COLI_INFECTION | 2.011598 | 0 | 0.026199 |
|  |  | KEGG_FC_GAMMA_R_MEDIATED_PHAGOCYTOSIS | 1.970646 | 0 | 0.023129 |
|  |  | KEGG_VIBRIO_CHOLERAE_INFECTION | 1.930018 | 0 | 0.0292 |
|  |  | KEGG_NOD_LIKE_RECEPTOR_SIGNALING_PATHWAY | 1.904647 | 0 | 0.033759 |
|  |  | KEGG_REGULATION_OF_ACTIN_CYTOSKELETON | 1.838353 | 0 | 0.076252 |
|  |  | KEGG_EPITHELIAL_CELL_SIGNALING_IN_HELICOBACTER_PYLORI_INFECTION | 1.834049 | 0 | 0.066558 |
|  |  | KEGG_NOTCH_SIGNALING_PATHWAY | 1.814429 | 0 | 0.052844 |
|  |  | KEGG_LONG_TERM_DEPRESSION | 1.794967 | 0 | 0.056163 |
|  |  | KEGG_RENAL_CELL_CARCINOMA | 1.780313 | 0 | 0.053608 |
|  |  | KEGG_CHEMOKINE_SIGNALING_PATHWAY | 1.818063 | 0.002151 | 0.062251 |
|  |  | KEGG_ENDOCYTOSIS | 1.760963 | 0.002165 | 0.050504 |
|  |  | KEGG_AXON_GUIDANCE | 1.82234 | 0.002398 | 0.066674 |
|  |  | KEGG_CYTOSOLIC_DNA_SENSING_PATHWAY | 1.789244 | 0.003717 | 0.054144 |
|  |  | KEGG_LEISHMANIA_INFECTION | 1.817425 | 0.004149 | 0.056207 |
|  |  | KEGG_CYTOKINE_CYTOKINE_RECEPTOR_INTERACTION | 1.772312 | 0.004545 | 0.054893 |
|  |  | KEGG_GAP_JUNCTION | 1.722632 | 0.00464 | 0.060257 |
|  |  | KEGG_MAPK_SIGNALING_PATHWAY | 1.71159 | 0.00464 | 0.063639 |
|  |  | KEGG_PATHWAYS_IN_CANCER | 1.694962 | 0.004785 | 0.068209 |
|  |  | KEGG_HYPERTROPHIC_CARDIOMYOPATHY_HCM | 1.661495 | 0.005 | 0.072406 |
|  |  | KEGG_CHRONIC_MYELOID_LEUKEMIA | 1.738444 | 0.006148 | 0.056719 |
|  |  | KEGG_T_CELL_RECEPTOR_SIGNALING_PATHWAY | 1.768759 | 0.006173 | 0.053607 |
|  |  | KEGG_LEUKOCYTE_TRANSENDOTHELIAL_MIGRATION | 1.807014 | 0.006682 | 0.051987 |
|  |  | KEGG_FOCAL_ADHESION | 1.744633 | 0.006865 | 0.056589 |
|  |  | KEGG_SPLICEOSOME | 1.761256 | 0.007813 | 0.053295 |
|  |  | KEGG_VEGF_SIGNALING_PATHWAY | 1.737269 | 0.008715 | 0.054599 |
|  |  | KEGG_PURINE_METABOLISM | 1.698277 | 0.008753 | 0.068521 |
| AC090921.1 | down-regulated | KEGG_PEROXISOME | -2.23786 | 0 | 0 |
|  |  | KEGG_GLYCINE_SERINE_AND_THREONINE_METABOLISM | -2.2003 | 0 | 0 |
|  |  | KEGG_PPAR_SIGNALING_PATHWAY | -2.14316 | 0 | 0 |
|  |  | KEGG_FATTY_ACID_METABOLISM | -2.13968 | 0 | 0 |
|  |  | KEGG_RETINOL_METABOLISM | -2.12742 | 0 | 0 |
|  |  | KEGG_PROPANOATE_METABOLISM | -2.11918 | 0 | 0 |
|  |  | KEGG_VALINE_LEUCINE_AND_ISOLEUCINE_DEGRADATION | -2.11804 | 0 | 0 |
|  |  | KEGG_DRUG_METABOLISM_CYTOCHROME_P450 | -2.09555 | 0 | 8.8E-05 |
|  |  | KEGG_TRYPTOPHAN_METABOLISM | -2.0641 | 0 | 0.000417 |
|  |  | KEGG_PRIMARY_BILE_ACID_BIOSYNTHESIS | -2.02888 | 0 | 0.000576 |
|  |  | KEGG_METABOLISM_OF_XENOBIOTICS_BY_CYTOCHROME_P450 | -1.97438 | 0 | 0.000986 |
|  |  | KEGG_HISTIDINE_METABOLISM | -1.94838 | 0 | 0.001361 |
|  |  | KEGG_BETA_ALANINE_METABOLISM | -1.93211 | 0 | 0.001563 |
|  |  | KEGG_ARGININE_AND_PROLINE_METABOLISM | -1.92092 | 0 | 0.001728 |
|  |  | KEGG_GLYOXYLATE_AND_DICARBOXYLATE_METABOLISM | -1.89649 | 0 | 0.002583 |
|  |  | KEGG_PYRUVATE_METABOLISM | -1.89208 | 0 | 0.002598 |
|  |  | KEGG_STEROID_HORMONE_BIOSYNTHESIS | -1.81757 | 0 | 0.006655 |
|  |  | KEGG_GLYCOLYSIS_GLUCONEOGENESIS | -1.77159 | 0.001681 | 0.010659 |
|  |  | KEGG_TYROSINE_METABOLISM | -1.99014 | 0.001704 | 0.000941 |
|  |  | KEGG_ALANINE_ASPARTATE_AND_GLUTAMATE_METABOLISM | -1.83674 | 0.001767 | 0.005359 |
|  |  | KEGG_BUTANOATE_METABOLISM | -2.04399 | 0.001887 | 0.000409 |
|  |  | KEGG_DRUG_METABOLISM_OTHER_ENZYMES | -1.74099 | 0.005319 | 0.013643 |
|  |  | KEGG_BIOSYNTHESIS_OF_UNSATURATED_FATTY_ACIDS | -1.80023 | 0.005376 | 0.008081 |
|  |  | KEGG_NITROGEN_METABOLISM | -1.6523 | 0.007987 | 0.029005 |
|  |  | KEGG_PHENYLALANINE_METABOLISM | -1.74227 | 0.009025 | 0.014004 |
| AC096637.2 | up-regulated | KEGG_SPLICEOSOME | 2.049564 | 0 | 0.009075 |
|  |  | KEGG_RNA_DEGRADATION | 1.903816 | 0 | 0.060135 |
|  |  | KEGG_NUCLEOTIDE_EXCISION_REPAIR | 1.882031 | 0 | 0.048437 |
|  |  | KEGG_PYRIMIDINE_METABOLISM | 1.880577 | 0 | 0.037525 |
|  |  | KEGG_DNA_REPLICATION | 1.845888 | 0 | 0.040732 |
|  |  | KEGG_HOMOLOGOUS_RECOMBINATION | 1.83372 | 0 | 0.03642 |
|  |  | KEGG_PURINE_METABOLISM | 1.760801 | 0 | 0.067346 |
|  |  | KEGG_BASE_EXCISION_REPAIR | 1.845686 | 0.002049 | 0.034913 |
|  |  | KEGG_CELL_CYCLE | 1.85095 | 0.00211 | 0.044872 |
|  |  | KEGG_UBIQUITIN_MEDIATED_PROTEOLYSIS | 1.786213 | 0.004073 | 0.060699 |
|  |  | KEGG_N_GLYCAN_BIOSYNTHESIS | 1.777646 | 0.00409 | 0.060044 |
|  |  | KEGG_RNA_POLYMERASE | 1.74126 | 0.005941 | 0.075859 |
|  |  | KEGG_BASAL_TRANSCRIPTION_FACTORS | 1.71824 | 0.008715 | 0.080693 |
|  |  | KEGG_OOCYTE_MEIOSIS | 1.681931 | 0.008909 | 0.100037 |
|  |  | KEGG_P53_SIGNALING_PATHWAY | 1.613719 | 0.008929 | 0.137533 |
|  |  | KEGG_PATHOGENIC_ESCHERICHIA_COLI_INFECTION | 1.661773 | 0.008969 | 0.113381 |
|  |  | KEGG_THYROID_CANCER | 1.683714 | 0.00907 | 0.104616 |
| AC096637.2 | down-regulated | KEGG_COMPLEMENT_AND_COAGULATION_CASCADES | -2.31454 | 0 | 0 |
|  |  | KEGG_TRYPTOPHAN_METABOLISM | -2.06601 | 0 | 0.000744 |
|  |  | KEGG_PRIMARY_BILE_ACID_BIOSYNTHESIS | -2.06367 | 0 | 0.000496 |
|  |  | KEGG_VALINE_LEUCINE_AND_ISOLEUCINE_DEGRADATION | -2.00551 | 0 | 0.000845 |
|  |  | KEGG_FATTY_ACID_METABOLISM | -2.00308 | 0 | 0.000676 |
|  |  | KEGG_DRUG_METABOLISM_CYTOCHROME_P450 | -1.98525 | 0 | 0.000781 |
|  |  | KEGG_RETINOL_METABOLISM | -1.9847 | 0 | 0.00067 |
|  |  | KEGG_PROPANOATE_METABOLISM | -1.97526 | 0 | 0.000632 |
|  |  | KEGG_PPAR_SIGNALING_PATHWAY | -1.77313 | 0.001669 | 0.016673 |
|  |  | KEGG_ARGININE_AND_PROLINE_METABOLISM | -1.7204 | 0.001689 | 0.024786 |
|  |  | KEGG_GLYCINE_SERINE_AND_THREONINE_METABOLISM | -1.83802 | 0.001855 | 0.009025 |
|  |  | KEGG_LINOLEIC_ACID_METABOLISM | -1.74172 | 0.003257 | 0.021753 |
|  |  | KEGG_STEROID_HORMONE_BIOSYNTHESIS | -1.79309 | 0.006568 | 0.016863 |
|  |  | KEGG_BUTANOATE_METABOLISM | -1.77524 | 0.007561 | 0.017553 |
|  |  | KEGG_PEROXISOME | -1.78786 | 0.009398 | 0.016428 |
| AP002478.1 | up-regulated | KEGG_PYRIMIDINE_METABOLISM | 1.977834 | 0 | 0.006284 |
|  |  | KEGG_RNA_POLYMERASE | 1.920554 | 0 | 0.013316 |
|  |  | KEGG_SPLICEOSOME | 1.820396 | 0 | 0.055237 |
|  |  | KEGG_RNA_DEGRADATION | 1.775087 | 0 | 0.06061 |
|  |  | KEGG_CELL_CYCLE | 1.774567 | 0 | 0.053463 |
|  |  | KEGG_PURINE_METABOLISM | 1.824442 | 0.002439 | 0.068206 |
|  |  | KEGG_PROTEASOME | 1.811267 | 0.004301 | 0.047555 |
|  |  | KEGG_HOMOLOGOUS_RECOMBINATION | 1.658839 | 0.00431 | 0.095298 |
|  |  | KEGG_NUCLEOTIDE_EXCISION_REPAIR | 1.681854 | 0.004329 | 0.092982 |
|  |  | KEGG_DNA_REPLICATION | 1.661344 | 0.006383 | 0.107997 |
|  |  | KEGG_PORPHYRIN_AND_CHLOROPHYLL_METABOLISM | 1.692788 | 0.007229 | 0.098283 |
|  |  | KEGG_AMINO_SUGAR_AND_NUCLEOTIDE_SUGAR_METABOLISM | 1.73506 | 0.007653 | 0.070535 |
|  |  | KEGG_GLUTATHIONE_METABOLISM | 1.691501 | 0.007937 | 0.090368 |
|  |  | KEGG_AMINOACYL_TRNA_BIOSYNTHESIS | 1.784459 | 0.009921 | 0.061991 |
| C10orf91 | up-regulated | KEGG_PURINE_METABOLISM | 2.013075 | 0 | 0.008171 |
|  |  | KEGG_PYRIMIDINE_METABOLISM | 2.010608 | 0 | 0.004086 |
|  |  | KEGG_SPLICEOSOME | 1.962463 | 0 | 0.009065 |
|  |  | KEGG_BASE_EXCISION_REPAIR | 1.940559 | 0 | 0.009583 |
|  |  | KEGG_RNA_DEGRADATION | 1.897867 | 0 | 0.016604 |
|  |  | KEGG_CELL_CYCLE | 1.873626 | 0 | 0.024353 |
|  |  | KEGG_HOMOLOGOUS_RECOMBINATION | 1.84875 | 0 | 0.02587 |
|  |  | KEGG_DNA_REPLICATION | 1.791002 | 0 | 0.046916 |
|  |  | KEGG_NUCLEOTIDE_EXCISION_REPAIR | 1.854818 | 0.002283 | 0.026636 |
|  |  | KEGG_UBIQUITIN_MEDIATED_PROTEOLYSIS | 1.78871 | 0.00237 | 0.043374 |
|  |  | KEGG_OOCYTE_MEIOSIS | 1.750023 | 0.002618 | 0.06385 |
|  |  | KEGG_P53_SIGNALING_PATHWAY | 1.691396 | 0.00277 | 0.076127 |
|  |  | KEGG_GLYCOSPHINGOLIPID_BIOSYNTHESIS_LACTO_AND_NEOLACTO_SERIES | 1.715512 | 0.003636 | 0.067162 |
|  |  | KEGG_RNA_POLYMERASE | 1.862954 | 0.004301 | 0.026569 |
|  |  | KEGG_BASAL_TRANSCRIPTION_FACTORS | 1.74022 | 0.004739 | 0.066361 |
|  |  | KEGG_NOTCH_SIGNALING_PATHWAY | 1.734203 | 0.004785 | 0.066107 |
|  |  | KEGG_ENDOCYTOSIS | 1.720229 | 0.005025 | 0.067908 |
| C10orf91 | down-regulated | KEGG_COMPLEMENT_AND_COAGULATION_CASCADES | -2.11303 | 0 | 0 |
|  |  | KEGG_STEROID_HORMONE_BIOSYNTHESIS | -2.05323 | 0 | 0.000314 |
|  |  | KEGG_RETINOL_METABOLISM | -2.02563 | 0 | 0.00021 |
|  |  | KEGG_TRYPTOPHAN_METABOLISM | -1.99917 | 0 | 0.000157 |
|  |  | KEGG_FATTY_ACID_METABOLISM | -1.99886 | 0 | 0.000126 |
|  |  | KEGG_DRUG_METABOLISM_CYTOCHROME_P450 | -1.99762 | 0 | 0.000105 |
|  |  | KEGG_PRIMARY_BILE_ACID_BIOSYNTHESIS | -1.94578 | 0 | 0.000573 |
|  |  | KEGG_VALINE_LEUCINE_AND_ISOLEUCINE_DEGRADATION | -1.91565 | 0 | 0.000911 |
|  |  | KEGG_METABOLISM_OF_XENOBIOTICS_BY_CYTOCHROME_P450 | -1.84822 | 0 | 0.002624 |
|  |  | KEGG_PPAR_SIGNALING_PATHWAY | -1.86356 | 0.001563 | 0.002347 |
|  |  | KEGG_GLYCINE_SERINE_AND_THREONINE_METABOLISM | -1.88604 | 0.003623 | 0.002001 |
|  |  | KEGG_PEROXISOME | -1.83614 | 0.005455 | 0.002971 |
|  |  | KEGG_PROPANOATE_METABOLISM | -1.78926 | 0.007299 | 0.005718 |
|  |  | KEGG_BETA_ALANINE_METABOLISM | -1.7706 | 0.007477 | 0.00649 |
|  |  | KEGG_BUTANOATE_METABOLISM | -1.77431 | 0.009124 | 0.006522 |
|  |  | KEGG_DRUG_METABOLISM_OTHER_ENZYMES | -1.62087 | 0.00982 | 0.034606 |
| LINC01116 | up-regulated | KEGG_PATHOGENIC_ESCHERICHIA_COLI_INFECTION | 1.936834 | 0 | 0.044302 |
|  |  | KEGG_VIBRIO_CHOLERAE_INFECTION | 1.912425 | 0 | 0.030174 |
|  |  | KEGG_RNA_POLYMERASE | 1.891705 | 0 | 0.034324 |
|  |  | KEGG_PURINE_METABOLISM | 1.879221 | 0 | 0.029941 |
|  |  | KEGG_GAP_JUNCTION | 1.843921 | 0 | 0.036542 |
|  |  | KEGG_AMINOACYL_TRNA_BIOSYNTHESIS | 1.830858 | 0 | 0.036184 |
|  |  | KEGG_PYRIMIDINE_METABOLISM | 1.808748 | 0 | 0.039855 |
|  |  | KEGG_AXON_GUIDANCE | 1.806509 | 0 | 0.03535 |
|  |  | KEGG_SPLICEOSOME | 1.801912 | 0 | 0.033605 |
|  |  | KEGG_FC_GAMMA_R_MEDIATED_PHAGOCYTOSIS | 1.789404 | 0 | 0.031848 |
|  |  | KEGG_REGULATION_OF_ACTIN_CYTOSKELETON | 1.76695 | 0 | 0.034566 |
|  |  | KEGG_NOTCH_SIGNALING_PATHWAY | 1.758556 | 0 | 0.035349 |
|  |  | KEGG_ENDOCYTOSIS | 1.742202 | 0 | 0.038279 |
|  |  | KEGG_PATHWAYS_IN_CANCER | 1.741993 | 0 | 0.036202 |
|  |  | KEGG_BLADDER_CANCER | 1.741766 | 0 | 0.034903 |
|  |  | KEGG_MAPK_SIGNALING_PATHWAY | 1.727053 | 0 | 0.038428 |
|  |  | KEGG_AMYOTROPHIC_LATERAL_SCLEROSIS_ALS | 1.560878 | 0 | 0.062031 |
|  |  | KEGG_RNA_DEGRADATION | 1.611637 | 0.002198 | 0.050555 |
|  |  | KEGG_UBIQUITIN_MEDIATED_PROTEOLYSIS | 1.626579 | 0.002283 | 0.049382 |
|  |  | KEGG_SNARE_INTERACTIONS_IN_VESICULAR_TRANSPORT | 1.60956 | 0.002353 | 0.049049 |
|  |  | KEGG_PHOSPHATIDYLINOSITOL_SIGNALING_SYSTEM | 1.708671 | 0.00241 | 0.0384 |
|  |  | KEGG_OOCYTE_MEIOSIS | 1.720004 | 0.002439 | 0.037846 |
|  |  | KEGG_EPITHELIAL_CELL_SIGNALING_IN_HELICOBACTER_PYLORI_INFECTION | 1.798734 | 0.002445 | 0.031214 |
|  |  | KEGG_NOD_LIKE_RECEPTOR_SIGNALING_PATHWAY | 1.736398 | 0.002445 | 0.035983 |
|  |  | KEGG_VASOPRESSIN_REGULATED_WATER_REABSORPTION | 1.747209 | 0.002457 | 0.038225 |
|  |  | KEGG_FOCAL_ADHESION | 1.726635 | 0.002632 | 0.036939 |
|  |  | KEGG_WNT_SIGNALING_PATHWAY | 1.636822 | 0.002688 | 0.045551 |
|  |  | KEGG_CYTOSOLIC_DNA_SENSING_PATHWAY | 1.781007 | 0.004405 | 0.031699 |
|  |  | KEGG_RENAL_CELL_CARCINOMA | 1.686594 | 0.004751 | 0.035227 |
|  |  | KEGG_NEUROTROPHIN_SIGNALING_PATHWAY | 1.687373 | 0.004796 | 0.036055 |
|  |  | KEGG_SMALL_CELL_LUNG_CANCER | 1.678429 | 0.004963 | 0.037563 |
|  |  | KEGG_LEISHMANIA_INFECTION | 1.779345 | 0.005013 | 0.029651 |
|  |  | KEGG_VEGF_SIGNALING_PATHWAY | 1.701801 | 0.005277 | 0.033341 |
|  |  | KEGG_PRION_DISEASES | 1.706273 | 0.005348 | 0.035274 |
|  |  | KEGG_LONG_TERM_DEPRESSION | 1.524812 | 0.0059 | 0.068635 |
|  |  | KEGG_DILATED_CARDIOMYOPATHY | 1.70263 | 0.006536 | 0.034149 |
|  |  | KEGG_CELL_CYCLE | 1.707139 | 0.006726 | 0.036191 |
|  |  | KEGG_NUCLEOTIDE_EXCISION_REPAIR | 1.645652 | 0.008889 | 0.043398 |
|  |  | KEGG_BASAL_CELL_CARCINOMA | 1.665068 | 0.009494 | 0.041347 |
|  |  | KEGG_GLYCOSPHINGOLIPID_BIOSYNTHESIS_GANGLIO_SERIES | 1.708349 | 0.009926 | 0.037049 |
|  |  | KEGG_T_CELL_RECEPTOR_SIGNALING_PATHWAY | 1.705404 | 0.009926 | 0.034128 |
| LINC01116 | down-regulated | KEGG_GLYCINE_SERINE_AND_THREONINE_METABOLISM | -2.09571 | 0 | 0.000333 |
|  |  | KEGG_PPAR_SIGNALING_PATHWAY | -1.7987 | 0.001597 | 0.024545 |
|  |  | KEGG_PRIMARY_BILE_ACID_BIOSYNTHESIS | -1.74475 | 0.001795 | 0.022878 |
|  |  | KEGG_FATTY_ACID_METABOLISM | -1.86579 | 0.001815 | 0.013783 |
|  |  | KEGG_BIOSYNTHESIS_OF_UNSATURATED_FATTY_ACIDS | -1.74817 | 0.00346 | 0.026261 |
|  |  | KEGG_RETINOL_METABOLISM | -1.75732 | 0.006279 | 0.029855 |
|  |  | KEGG_PEROXISOME | -1.71149 | 0.009174 | 0.030254 |
| LINC01224 | up-regulated | KEGG_PROGESTERONE_MEDIATED_OOCYTE_MATURATION | 2.094223 | 0 | 0.010182 |
|  |  | KEGG_CELL_CYCLE | 2.091396 | 0 | 0.005091 |
|  |  | KEGG_NUCLEOTIDE_EXCISION_REPAIR | 2.053364 | 0 | 0.006766 |
|  |  | KEGG_N_GLYCAN_BIOSYNTHESIS | 2.021683 | 0 | 0.007419 |
|  |  | KEGG_OOCYTE_MEIOSIS | 2.008925 | 0 | 0.006684 |
|  |  | KEGG_SPLICEOSOME | 1.98771 | 0 | 0.009367 |
|  |  | KEGG_RNA_DEGRADATION | 1.983876 | 0 | 0.008558 |
|  |  | KEGG_UBIQUITIN_MEDIATED_PROTEOLYSIS | 1.980445 | 0 | 0.007775 |
|  |  | KEGG_DNA_REPLICATION | 1.979186 | 0 | 0.006911 |
|  |  | KEGG_BASE_EXCISION_REPAIR | 1.971122 | 0 | 0.006303 |
|  |  | KEGG_RIBOFLAVIN_METABOLISM | 1.962573 | 0 | 0.00619 |
|  |  | KEGG_THYROID_CANCER | 1.939003 | 0 | 0.00733 |
|  |  | KEGG_PYRIMIDINE_METABOLISM | 1.92194 | 0 | 0.007578 |
|  |  | KEGG_MISMATCH_REPAIR | 1.913905 | 0 | 0.00715 |
|  |  | KEGG_BASAL_TRANSCRIPTION_FACTORS | 1.888832 | 0 | 0.009195 |
|  |  | KEGG_PURINE_METABOLISM | 1.887997 | 0 | 0.008654 |
|  |  | KEGG_PANCREATIC_CANCER | 1.852109 | 0 | 0.011238 |
|  |  | KEGG_PATHOGENIC_ESCHERICHIA_COLI_INFECTION | 1.830898 | 0 | 0.01369 |
|  |  | KEGG_RNA_POLYMERASE | 1.809341 | 0 | 0.01691 |
|  |  | KEGG_MTOR_SIGNALING_PATHWAY | 1.75982 | 0 | 0.025348 |
|  |  | KEGG_TIGHT_JUNCTION | 1.67553 | 0 | 0.040273 |
|  |  | KEGG_MELANOMA | 1.615638 | 0 | 0.051688 |
|  |  | KEGG_HOMOLOGOUS_RECOMBINATION | 1.954977 | 0.00207 | 0.006146 |
|  |  | KEGG_NON_SMALL_CELL_LUNG_CANCER | 1.854821 | 0.002174 | 0.0117 |
|  |  | KEGG_PROSTATE_CANCER | 1.747554 | 0.002299 | 0.025984 |
|  |  | KEGG_BLADDER_CANCER | 1.765857 | 0.002375 | 0.02445 |
|  |  | KEGG_INOSITOL_PHOSPHATE_METABOLISM | 1.717247 | 0.004329 | 0.029607 |
|  |  | KEGG_ADHERENS_JUNCTION | 1.753127 | 0.004396 | 0.025883 |
|  |  | KEGG_RENAL_CELL_CARCINOMA | 1.767797 | 0.004405 | 0.025063 |
|  |  | KEGG_ENDOCYTOSIS | 1.739223 | 0.004425 | 0.028273 |
|  |  | KEGG_TGF_BETA_SIGNALING_PATHWAY | 1.731662 | 0.004435 | 0.027834 |
|  |  | KEGG_WNT_SIGNALING_PATHWAY | 1.784296 | 0.004902 | 0.021351 |
|  |  | KEGG_GAP_JUNCTION | 1.669264 | 0.004902 | 0.039077 |
|  |  | KEGG_PATHWAYS_IN_CANCER | 1.730283 | 0.004914 | 0.027416 |
|  |  | KEGG_AMINOACYL_TRNA_BIOSYNTHESIS | 1.808777 | 0.00611 | 0.016142 |
|  |  | KEGG_COLORECTAL_CANCER | 1.732447 | 0.006466 | 0.02847 |
|  |  | KEGG_LONG_TERM_POTENTIATION | 1.752356 | 0.006993 | 0.025039 |
|  |  | KEGG_SELENOAMINO_ACID_METABOLISM | 1.689726 | 0.008493 | 0.037518 |
|  |  | KEGG_NEUROTROPHIN_SIGNALING_PATHWAY | 1.711726 | 0.008602 | 0.030778 |
|  |  | KEGG_VASOPRESSIN_REGULATED_WATER_REABSORPTION | 1.684717 | 0.008753 | 0.037911 |
|  |  | KEGG_EPITHELIAL_CELL_SIGNALING_IN_HELICOBACTER_PYLORI_INFECTION | 1.61944 | 0.008969 | 0.053386 |
| LINC01224 | down-regulated | KEGG_COMPLEMENT_AND_COAGULATION_CASCADES | -2.2417 | 0 | 0 |
|  |  | KEGG_TRYPTOPHAN_METABOLISM | -1.86612 | 0.001664 | 0.021398 |
|  |  | KEGG_PRIMARY_BILE_ACID_BIOSYNTHESIS | -1.84042 | 0.001828 | 0.021118 |
| MAFG-DT | up-regulated | KEGG_PYRIMIDINE_METABOLISM | 1.8948 | 0 | 0.030786 |
|  |  | KEGG_SPLICEOSOME | 1.881264 | 0 | 0.025569 |
|  |  | KEGG_RNA_POLYMERASE | 1.865848 | 0 | 0.025692 |
|  |  | KEGG_PURINE_METABOLISM | 1.832061 | 0 | 0.034194 |
|  |  | KEGG_HOMOLOGOUS_RECOMBINATION | 1.797076 | 0 | 0.04541 |
|  |  | KEGG_DNA_REPLICATION | 1.788152 | 0 | 0.037129 |
|  |  | KEGG_VIBRIO_CHOLERAE_INFECTION | 1.73276 | 0 | 0.04889 |
|  |  | KEGG_RIBOSOME | 1.727377 | 0.002062 | 0.04825 |
|  |  | KEGG_RNA_DEGRADATION | 1.738594 | 0.002137 | 0.049304 |
|  |  | KEGG_PROTEASOME | 1.916197 | 0.002179 | 0.034672 |
|  |  | KEGG_CELL_CYCLE | 1.814042 | 0.002273 | 0.041179 |
|  |  | KEGG_HUNTINGTONS_DISEASE | 1.920193 | 0.002299 | 0.068227 |
|  |  | KEGG_AMINOACYL_TRNA_BIOSYNTHESIS | 1.793597 | 0.004175 | 0.042526 |
|  |  | KEGG_NUCLEOTIDE_EXCISION_REPAIR | 1.690839 | 0.004357 | 0.061308 |
|  |  | KEGG_ALZHEIMERS_DISEASE | 1.789917 | 0.007481 | 0.040036 |
|  |  | KEGG_VASOPRESSIN_REGULATED_WATER_REABSORPTION | 1.657387 | 0.008889 | 0.079509 |
| MAFG-DT | down-regulated | KEGG_COMPLEMENT_AND_COAGULATION_CASCADES | -2.06483 | 0 | 0 |
|  |  | KEGG_RETINOL_METABOLISM | -1.91446 | 0 | 0.003246 |
|  |  | KEGG_GLYCINE_SERINE_AND_THREONINE_METABOLISM | -1.86107 | 0 | 0.006263 |
|  |  | KEGG_PPAR_SIGNALING_PATHWAY | -1.81447 | 0 | 0.011765 |
|  |  | KEGG_TRYPTOPHAN_METABOLISM | -1.75684 | 0.00155 | 0.021631 |
|  |  | KEGG_FATTY_ACID_METABOLISM | -1.77896 | 0.001712 | 0.018599 |
|  |  | KEGG_LINOLEIC_ACID_METABOLISM | -1.61406 | 0.004208 | 0.080771 |
|  |  | KEGG_STARCH_AND_SUCROSE_METABOLISM | -1.64268 | 0.009009 | 0.070062 |
| SERTAD4-AS1 | up-regulated | KEGG_ECM_RECEPTOR_INTERACTION | 1.934583 | 0 | 0.016847 |
|  |  | KEGG_DILATED_CARDIOMYOPATHY | 1.820891 | 0 | 0.065161 |
|  |  | KEGG_VASCULAR_SMOOTH_MUSCLE_CONTRACTION | 1.814054 | 0 | 0.046718 |
|  |  | KEGG_FOCAL_ADHESION | 1.761864 | 0 | 0.083427 |
|  |  | KEGG_HYPERTROPHIC_CARDIOMYOPATHY_HCM | 1.742862 | 0 | 0.083672 |
|  |  | KEGG_ARRHYTHMOGENIC_RIGHT_VENTRICULAR_CARDIOMYOPATHY_ARVC | 1.69881 | 0 | 0.107788 |
|  |  | KEGG_NEUROACTIVE_LIGAND_RECEPTOR_INTERACTION | 1.615974 | 0.001198 | 0.159074 |
|  |  | KEGG_CALCIUM_SIGNALING_PATHWAY | 1.687716 | 0.001372 | 0.106964 |
|  |  | KEGG_CELL_ADHESION_MOLECULES_CAMS | 1.687452 | 0.00487 | 0.09544 |
|  |  | KEGG_LEUKOCYTE_TRANSENDOTHELIAL_MIGRATION | 1.664273 | 0.004934 | 0.112461 |
|  |  | KEGG_ETHER_LIPID_METABOLISM | 1.571244 | 0.007407 | 0.175222 |
|  |  | KEGG_TGF_BETA_SIGNALING_PATHWAY | 1.648072 | 0.008403 | 0.121672 |
