# Supplementary material for: A novel molecular-clinicopathologic nomogram to improve prognosis prediction of hepatocellular carcinoma
Source: Aging (Albany NY). 2020 Jun 30;12(13):12896–920. doi: 10.18632/aging.103350 (PMC7377850; doi:10.18632/aging.103350)
Supplement: Supplementary Table 8 [file aging-12-103350-s002..docx]

**Supplementary Table 8.Gene sets enriched in recurrence related classifier**

| **Gene** | **Phenotype** | **Gene Set** | **NES** | **NOM p-val** | **FDR q-val** |
| --- | --- | --- | --- | --- | --- |
| AC004477.1 | up-regulated | KEGG_PYRIMIDINE_METABOLISM | 1.882411 | 0 | 0.1433332 |
|  |  | KEGG_SPLICEOSOME | 1.814374 | 0.002024292 | 0.08482 |
|  |  | KEGG_NUCLEOTIDE_EXCISION_REPAIR | 1.843232 | 0.002066116 | 0.1184254 |
|  |  | KEGG_GLYCOSYLPHOSPHATIDYLINOSITOL_GPI_ANCHOR_BIOSYNTHESIS | 1.749359 | 0.002079002 | 0.143701 |
|  |  | KEGG_BASE_EXCISION_REPAIR | 1.815455 | 0.00210084 | 0.1114659 |
| AC004477.1 | down-regulated | KEGG_COMPLEMENT_AND_COAGULATION_CASCADES | -2.24697 | 0 | 0 |
| AC010307.4 | up-regulated | KEGG_ALZHEIMERS_DISEASE | 1.775944 | 0.005102041 | 0.2053707 |
|  |  | KEGG_FRUCTOSE_AND_MANNOSE_METABOLISM | 1.690693 | 0.005970149 | 0.1671946 |
|  |  | KEGG_OTHER_GLYCAN_DEGRADATION | 1.706024 | 0.006160164 | 0.1721993 |
|  |  | KEGG_PORPHYRIN_AND_CHLOROPHYLL_METABOLISM | 1.752648 | 0.007894737 | 0.1723269 |
| AC010307.4 | down-regulated | KEGG_COMPLEMENT_AND_COAGULATION_CASCADES | -1.89994 | 0 | 0.0184316 |
| AC034229.4 | up-regulated | KEGG_SPLICEOSOME | 1.854351 | 0 | 0.1419404 |
|  |  | KEGG_CELL_CYCLE | 1.822007 | 0 | 0.0984223 |
|  |  | KEGG_HOMOLOGOUS_RECOMBINATION | 1.817151 | 0 | 0.0729155 |
|  |  | KEGG_GLYCOSPHINGOLIPID_BIOSYNTHESIS_LACTO_AND_NEOLACTO_SERIES | 1.76655 | 0 | 0.1004111 |
|  |  | KEGG_RNA_POLYMERASE | 1.698331 | 0.002070393 | 0.1013296 |
|  |  | KEGG_NUCLEOTIDE_EXCISION_REPAIR | 1.739976 | 0.002109705 | 0.1079069 |
|  |  | KEGG_DNA_REPLICATION | 1.738834 | 0.002136752 | 0.0907494 |
|  |  | KEGG_PYRIMIDINE_METABOLISM | 1.720833 | 0.004444445 | 0.0930357 |
|  |  | KEGG_RNA_DEGRADATION | 1.678405 | 0.008179959 | 0.0991225 |
| AC034229.4 | down-regulated | KEGG_COMPLEMENT_AND_COAGULATION_CASCADES | -2.04638 | 0 | 0 |
|  |  | KEGG_TRYPTOPHAN_METABOLISM | -2.04614 | 0 | 0 |
|  |  | KEGG_PRIMARY_BILE_ACID_BIOSYNTHESIS | -2.03388 | 0 | 0 |
|  |  | KEGG_VALINE_LEUCINE_AND_ISOLEUCINE_DEGRADATION | -2.00463 | 0 | 0.000501 |
|  |  | KEGG_DRUG_METABOLISM_CYTOCHROME_P450 | -1.95473 | 0 | 0.0018473 |
|  |  | KEGG_PROPANOATE_METABOLISM | -1.93866 | 0 | 0.00175 |
|  |  | KEGG_RETINOL_METABOLISM | -1.92605 | 0 | 0.0023583 |
|  |  | KEGG_CITRATE_CYCLE_TCA_CYCLE | -1.89581 | 0 | 0.0032855 |
|  |  | KEGG_PPAR_SIGNALING_PATHWAY | -1.81554 | 0 | 0.0104087 |
|  |  | KEGG_BUTANOATE_METABOLISM | -1.81061 | 0 | 0.0107841 |
|  |  | KEGG_STEROID_HORMONE_BIOSYNTHESIS | -1.83001 | 0.003025719 | 0.0092228 |
|  |  | KEGG_FATTY_ACID_METABOLISM | -1.83635 | 0.003831418 | 0.0091399 |
|  |  | KEGG_ARGININE_AND_PROLINE_METABOLISM | -1.66814 | 0.004601227 | 0.0468841 |
|  |  | KEGG_METABOLISM_OF_XENOBIOTICS_BY_CYTOCHROME_P450 | -1.80636 | 0.004807693 | 0.0106383 |
|  |  | KEGG_ALANINE_ASPARTATE_AND_GLUTAMATE_METABOLISM | -1.71955 | 0.004823151 | 0.0279778 |
|  |  | KEGG_GLYCOLYSIS_GLUCONEOGENESIS | -1.67149 | 0.00610687 | 0.0479551 |
|  |  | KEGG_PEROXISOME | -1.73822 | 0.009259259 | 0.0250589 |
| CDKN2A-DT | up-regulated | KEGG_PYRIMIDINE_METABOLISM | 2.161186 | 0 | 0 |
|  |  | KEGG_BASE_EXCISION_REPAIR | 2.151221 | 0 | 0 |
|  |  | KEGG_DNA_REPLICATION | 2.007602 | 0 | 0.0086466 |
|  |  | KEGG_CELL_CYCLE | 1.967839 | 0 | 0.0094977 |
|  |  | KEGG_MISMATCH_REPAIR | 1.890775 | 0.001976285 | 0.0191223 |
|  |  | KEGG_RNA_POLYMERASE | 1.881427 | 0.002164502 | 0.0188305 |
|  |  | KEGG_P53_SIGNALING_PATHWAY | 1.784138 | 0.002169197 | 0.0436388 |
|  |  | KEGG_PURINE_METABOLISM | 1.774269 | 0.002293578 | 0.0448885 |
|  |  | KEGG_HOMOLOGOUS_RECOMBINATION | 1.868393 | 0.00409836 | 0.0192727 |
|  |  | KEGG_NUCLEOTIDE_EXCISION_REPAIR | 1.797687 | 0.004192872 | 0.0411566 |
|  |  | KEGG_RNA_DEGRADATION | 1.738917 | 0.00422833 | 0.0563846 |
|  |  | KEGG_ONE_CARBON_POOL_BY_FOLATE | 1.740197 | 0.006 | 0.0609137 |
| CDKN2B-AS1 | up-regulated | KEGG_BASE_EXCISION_REPAIR | 2.157417 | 0 | 0 |
|  |  | KEGG_CELL_CYCLE | 2.025484 | 0 | 0.0053302 |
|  |  | KEGG_DNA_REPLICATION | 2.022618 | 0 | 0.0035534 |
|  |  | KEGG_PYRIMIDINE_METABOLISM | 2.002849 | 0 | 0.0065632 |
|  |  | KEGG_MISMATCH_REPAIR | 1.932552 | 0 | 0.017711 |
|  |  | KEGG_NUCLEOTIDE_EXCISION_REPAIR | 1.871242 | 0 | 0.0229242 |
|  |  | KEGG_OOCYTE_MEIOSIS | 1.798658 | 0 | 0.0466668 |
|  |  | KEGG_PURINE_METABOLISM | 1.725546 | 0 | 0.0766891 |
|  |  | KEGG_HOMOLOGOUS_RECOMBINATION | 1.891719 | 0.002070393 | 0.0217981 |
|  |  | KEGG_RNA_DEGRADATION | 1.682994 | 0.004048583 | 0.1072478 |
|  |  | KEGG_SPLICEOSOME | 1.763667 | 0.004210526 | 0.0610589 |
|  |  | KEGG_P53_SIGNALING_PATHWAY | 1.731925 | 0.007194245 | 0.0788217 |
|  |  | KEGG_PROGESTERONE_MEDIATED_OOCYTE_MATURATION | 1.67154 | 0.00954654 | 0.1015228 |
| CDKN2B-AS1 | down-regulated | KEGG_COMPLEMENT_AND_COAGULATION_CASCADES | -1.82942 | 0.001912046 | 0.0994761 |
| FIRRE | up-regulated | KEGG_SELENOAMINO_ACID_METABOLISM | 2.030367 | 0 | 0.0133311 |
|  |  | KEGG_UBIQUITIN_MEDIATED_PROTEOLYSIS | 1.969766 | 0 | 0.0084448 |
|  |  | KEGG_OOCYTE_MEIOSIS | 1.925874 | 0 | 0.0163465 |
|  |  | KEGG_RNA_DEGRADATION | 1.913219 | 0 | 0.013821 |
|  |  | KEGG_CELL_CYCLE | 1.835872 | 0 | 0.0322219 |
|  |  | KEGG_LONG_TERM_POTENTIATION | 1.822678 | 0 | 0.0367409 |
|  |  | KEGG_SPLICEOSOME | 1.799989 | 0 | 0.044392 |
|  |  | KEGG_NUCLEOTIDE_EXCISION_REPAIR | 1.731312 | 0 | 0.0555017 |
|  |  | KEGG_NON_SMALL_CELL_LUNG_CANCER | 1.726698 | 0 | 0.0555348 |
|  |  | KEGG_GNRH_SIGNALING_PATHWAY | 1.718594 | 0 | 0.0542263 |
|  |  | KEGG_INOSITOL_PHOSPHATE_METABOLISM | 1.670016 | 0 | 0.0679511 |
|  |  | KEGG_MTOR_SIGNALING_PATHWAY | 1.657405 | 0 | 0.0647696 |
|  |  | KEGG_HOMOLOGOUS_RECOMBINATION | 1.756401 | 0.002141328 | 0.0626013 |
|  |  | KEGG_BASE_EXCISION_REPAIR | 1.731401 | 0.002188184 | 0.0587934 |
|  |  | KEGG_AMINOACYL_TRNA_BIOSYNTHESIS | 1.752395 | 0.002202643 | 0.0552516 |
|  |  | KEGG_RENAL_CELL_CARCINOMA | 1.854815 | 0.002267574 | 0.0286817 |
|  |  | KEGG_BASAL_TRANSCRIPTION_FACTORS | 1.776153 | 0.002288329 | 0.0537365 |
|  |  | KEGG_VASOPRESSIN_REGULATED_WATER_REABSORPTION | 1.725041 | 0.002304148 | 0.0532952 |
|  |  | KEGG_PROGESTERONE_MEDIATED_OOCYTE_MATURATION | 1.810832 | 0.002364066 | 0.0401244 |
|  |  | KEGG_PYRIMIDINE_METABOLISM | 1.745637 | 0.002475248 | 0.0519995 |
|  |  | KEGG_INSULIN_SIGNALING_PATHWAY | 1.716026 | 0.0025 | 0.0528862 |
|  |  | KEGG_VEGF_SIGNALING_PATHWAY | 1.664825 | 0.002538071 | 0.0688925 |
|  |  | KEGG_PURINE_METABOLISM | 1.755876 | 0.002610966 | 0.057681 |
|  |  | KEGG_GAP_JUNCTION | 1.684191 | 0.002617801 | 0.0656244 |
|  |  | KEGG_NEUROTROPHIN_SIGNALING_PATHWAY | 1.749819 | 0.004694836 | 0.0522481 |
|  |  | KEGG_GLIOMA | 1.661296 | 0.004889976 | 0.0681881 |
|  |  | KEGG_AMYOTROPHIC_LATERAL_SCLEROSIS_ALS | 1.560686 | 0.006410257 | 0.0801134 |
|  |  | KEGG_LONG_TERM_DEPRESSION | 1.585483 | 0.008241759 | 0.0725313 |
|  |  | KEGG_MISMATCH_REPAIR | 1.695874 | 0.008403362 | 0.0607963 |
|  |  | KEGG_RIG_I_LIKE_RECEPTOR_SIGNALING_PATHWAY | 1.634719 | 0.008583691 | 0.0729222 |
|  |  | KEGG_VIBRIO_CHOLERAE_INFECTION | 1.620923 | 0.009049774 | 0.0727484 |
| LINC01572 | up-regulated | KEGG_RNA_DEGRADATION | 1.987484 | 0 | 0.0260703 |
|  |  | KEGG_CELL_CYCLE | 1.976948 | 0 | 0.015683 |
|  |  | KEGG_HOMOLOGOUS_RECOMBINATION | 1.895635 | 0 | 0.0528617 |
|  |  | KEGG_P53_SIGNALING_PATHWAY | 1.891119 | 0 | 0.0425299 |
|  |  | KEGG_UBIQUITIN_MEDIATED_PROTEOLYSIS | 1.849482 | 0 | 0.0578978 |
|  |  | KEGG_SELENOAMINO_ACID_METABOLISM | 1.814316 | 0 | 0.0713063 |
|  |  | KEGG_BLADDER_CANCER | 1.785293 | 0 | 0.0673514 |
|  |  | KEGG_SPLICEOSOME | 1.779974 | 0 | 0.0655134 |
|  |  | KEGG_PURINE_METABOLISM | 1.713242 | 0 | 0.0704664 |
|  |  | KEGG_BASAL_TRANSCRIPTION_FACTORS | 1.750618 | 0.002109705 | 0.0744162 |
|  |  | KEGG_PYRIMIDINE_METABOLISM | 1.775914 | 0.00212766 | 0.0619008 |
|  |  | KEGG_INSULIN_SIGNALING_PATHWAY | 1.808781 | 0.002150538 | 0.0665445 |
|  |  | KEGG_NON_SMALL_CELL_LUNG_CANCER | 1.720886 | 0.004310345 | 0.0816658 |
|  |  | KEGG_OOCYTE_MEIOSIS | 1.748819 | 0.004494382 | 0.0696383 |
|  |  | KEGG_CYSTEINE_AND_METHIONINE_METABOLISM | 1.807352 | 0.006410257 | 0.0599104 |
|  |  | KEGG_GLYCOSAMINOGLYCAN_DEGRADATION | 1.713885 | 0.006479482 | 0.0740931 |
|  |  | KEGG_PROGESTERONE_MEDIATED_OOCYTE_MATURATION | 1.724483 | 0.006521739 | 0.08552 |
|  |  | KEGG_ENDOMETRIAL_CANCER | 1.714057 | 0.008510638 | 0.0779166 |
|  |  | KEGG_RENAL_CELL_CARCINOMA | 1.712633 | 0.008528785 | 0.0676303 |
|  |  | KEGG_MTOR_SIGNALING_PATHWAY | 1.663535 | 0.008639309 | 0.0778671 |
|  |  | KEGG_SNARE_INTERACTIONS_IN_VESICULAR_TRANSPORT | 1.714904 | 0.008658009 | 0.0819649 |
|  |  | KEGG_COLORECTAL_CANCER | 1.660958 | 0.008849558 | 0.077446 |
| MAFG-DT | up-regulated | KEGG_PROTEASOME | 1.898511 | 0 | 0.0646525 |
|  |  | KEGG_DNA_REPLICATION | 1.884555 | 0 | 0.0461328 |
|  |  | KEGG_HOMOLOGOUS_RECOMBINATION | 1.86804 | 0 | 0.041212 |
|  |  | KEGG_PATHOGENIC_ESCHERICHIA_COLI_INFECTION | 1.866164 | 0 | 0.030909 |
|  |  | KEGG_EPITHELIAL_CELL_SIGNALING_IN_HELICOBACTER_PYLORI_INFECTION | 1.833576 | 0 | 0.0397287 |
|  |  | KEGG_PYRIMIDINE_METABOLISM | 1.829 | 0 | 0.034808 |
|  |  | KEGG_PURINE_METABOLISM | 1.802488 | 0 | 0.0372259 |
|  |  | KEGG_RNA_DEGRADATION | 1.723103 | 0 | 0.059731 |
|  |  | KEGG_LYSOSOME | 1.753442 | 0.001754386 | 0.051078 |
|  |  | KEGG_CELL_CYCLE | 1.813858 | 0.001855288 | 0.036316 |
|  |  | KEGG_SPLICEOSOME | 1.785194 | 0.001912046 | 0.0432764 |
|  |  | KEGG_BASE_EXCISION_REPAIR | 1.737421 | 0.001919386 | 0.0581026 |
|  |  | KEGG_VIBRIO_CHOLERAE_INFECTION | 1.728294 | 0.003418803 | 0.0597117 |
|  |  | KEGG_AMYOTROPHIC_LATERAL_SCLEROSIS_ALS | 1.610554 | 0.004694836 | 0.1271549 |
|  |  | KEGG_GLYCEROPHOSPHOLIPID_METABOLISM | 1.553804 | 0.006472492 | 0.1610282 |
|  |  | KEGG_NUCLEOTIDE_EXCISION_REPAIR | 1.759911 | 0.007462686 | 0.0530578 |
|  |  | KEGG_VASOPRESSIN_REGULATED_WATER_REABSORPTION | 1.660342 | 0.008944544 | 0.0903513 |
| MAFG-DT | down-regulated | KEGG_VALINE_LEUCINE_AND_ISOLEUCINE_DEGRADATION | -1.95117 | 0 | 0.0154007 |
|  |  | KEGG_COMPLEMENT_AND_COAGULATION_CASCADES | -1.94505 | 0 | 0.0077003 |
|  |  | KEGG_FATTY_ACID_METABOLISM | -1.86068 | 0 | 0.0114795 |
|  |  | KEGG_BUTANOATE_METABOLISM | -1.94397 | 0.004524887 | 0.0052307 |
|  |  | KEGG_BETA_ALANINE_METABOLISM | -1.76285 | 0.008714597 | 0.0259909 |
|  |  | KEGG_GLYCINE_SERINE_AND_THREONINE_METABOLISM | -1.88133 | 0.00886918 | 0.010562 |
| MIR9-3HG | up-regulated | KEGG_PROTEASOME | 1.947667 | 0 | 0.0240138 |
|  |  | KEGG_PATHOGENIC_ESCHERICHIA_COLI_INFECTION | 1.866901 | 0 | 0.0485872 |
|  |  | KEGG_PYRIMIDINE_METABOLISM | 1.854508 | 0 | 0.0368648 |
|  |  | KEGG_HOMOLOGOUS_RECOMBINATION | 1.852072 | 0 | 0.0282458 |
|  |  | KEGG_DNA_REPLICATION | 1.84711 | 0 | 0.0238547 |
|  |  | KEGG_EPITHELIAL_CELL_SIGNALING_IN_HELICOBACTER_PYLORI_INFECTION | 1.828278 | 0 | 0.0295346 |
|  |  | KEGG_SPLICEOSOME | 1.821867 | 0 | 0.0280236 |
|  |  | KEGG_PURINE_METABOLISM | 1.818004 | 0 | 0.0261228 |
|  |  | KEGG_CELL_CYCLE | 1.795767 | 0 | 0.0318865 |
|  |  | KEGG_RNA_DEGRADATION | 1.737275 | 0 | 0.0478777 |
|  |  | KEGG_VIBRIO_CHOLERAE_INFECTION | 1.74061 | 0.001736111 | 0.0501958 |
|  |  | KEGG_LYSOSOME | 1.73378 | 0.001848429 | 0.0464754 |
|  |  | KEGG_NUCLEOTIDE_EXCISION_REPAIR | 1.7477 | 0.001934236 | 0.0546717 |
|  |  | KEGG_VASOPRESSIN_REGULATED_WATER_REABSORPTION | 1.676043 | 0.003724395 | 0.0715414 |
|  |  | KEGG_BASE_EXCISION_REPAIR | 1.740956 | 0.005736138 | 0.0542815 |
|  |  | KEGG_MISMATCH_REPAIR | 1.704169 | 0.005859375 | 0.0595572 |
|  |  | KEGG_AMYOTROPHIC_LATERAL_SCLEROSIS_ALS | 1.625828 | 0.00761035 | 0.1027218 |
|  |  | KEGG_ENDOCYTOSIS | 1.685487 | 0.009090909 | 0.0689224 |
|  |  | KEGG_GLYCEROPHOSPHOLIPID_METABOLISM | 1.569313 | 0.009661836 | 0.1333598 |
|  |  | KEGG_UBIQUITIN_MEDIATED_PROTEOLYSIS | 1.705638 | 0.00984252 | 0.0623407 |
|  |  | KEGG_VALINE_LEUCINE_AND_ISOLEUCINE_DEGRADATION | -1.91771 | 0 | 0.0074307 |
| MIR9-3HG | down-regulated | KEGG_BUTANOATE_METABOLISM | -1.87478 | 0 | 0.013865 |
|  |  | KEGG_FATTY_ACID_METABOLISM | -1.86256 | 0.002087683 | 0.0107706 |
|  |  | KEGG_COMPLEMENT_AND_COAGULATION_CASCADES | -1.94241 | 0.002227172 | 0.0115371 |
|  |  | KEGG_GLYCINE_SERINE_AND_THREONINE_METABOLISM | -1.86406 | 0.004291846 | 0.0131682 |
