# Supplementary material for: A novel molecular-clinicopathologic nomogram to improve prognosis prediction of hepatocellular carcinoma
Source: Aging (Albany NY). 2020 Jun 30;12(13):12896–920. doi: 10.18632/aging.103350 (PMC7377850; doi:10.18632/aging.103350)
Supplement: Supplementary Table 10 [file aging-12-103350-s004..docx]

| **Supplementary Table 10. Correlations between risk score of the 8-lncRNA-based classifier with overall survival and clinicopathological characteristics in training cohort, test cohort, TCGA cohort and GEO cohort.** | | | | | |
| --- | --- | --- | --- | --- | --- |
| Clinicopathological variables | | Number of  patients | High risk | Low risk | P value |
| **Training Cohort** | Age |  |  |  |  |
|  | <65 | 95(60.9%) | 29 (30.5%) | 66 (69.5%) |  |
|  | >65 | 61(39.1%) | 15 (24.6%) | 46 (75.4%) | 0.321 |
|  | M |  |  |  |  |
|  | M0 | 120(97.6%) | 32(26.7%) | 88(73.3%) |  |
|  | M1 | 3(2.4%) | 0(0%) | 3(100%) | 0.298 |
|  | N |  |  |  |  |
|  | N0 | 115(98.9%) | 32(27.8%) | 83(72.2%) |  |
|  | N1 | 0(0%) |  |  |  |
|  | Stage |  |  |  |  |
|  | Stage I&II | 107(73.8%) | 28(26.2%) | 79(73.8%) |  |
|  | Stage III&IV | 38(26.2%) | 12(31.6%) | 26(68.4%) | 0.521 |
|  | T |  |  |  |  |
|  | T1&2 | 114(74.0%) | 31(27.2%) | 83(72.8%) |  |
|  | T3&4 | 40(26.0%) | 13(32.5%) | 27(67.5%) | 0.523 |
|  | Child pugh classification |  |  |  |  |
|  | A&B | 101(90.2%) | 24(23.8%) | 77(76.2%) |  |
|  | C&D | 11(9.8%) | 0(0%) | 11(100%) | 0.068 |
|  | Performance Status |  |  |  |  |
|  | Status 0 | 74(56.5%) | 18(24.3%) | 56(75.7%) |  |
|  | Status 1 | 40(30.5%) | 9(22.5%) | 31(77.5%) |  |
|  | Status 2 | 11(8.4%) | 2(18.2%) | 9(81.8%) |  |
|  | Status 3  Status 4 | 5(3.8%)  1(0.8%) | 3(60%)  1(100%) | 2(40%)  0(0%) | 0.155 |
|  | Family History |  |  |  |  |
|  | NO | 94(68.6%) | 27(28.7%) | 67(71.3%) |  |
|  | YES | 43(31.4%) | 12(27.9%) | 31(72.1%) | 0.922 |
|  | Grade |  |  |  |  |
|  | G1&2 | 94(60.6%) | 23(24.5%) | 71(75.5%) |  |
|  | G3&4 | 61(39.4%) | 21(34.4%) | 40(65.6%) | 0.179 |
|  | Adjacent hepatic tissue inflammation |  |  |  |  |
|  | None | 52(49.5%) | 12(23.1%) | 40(76.9%) |  |
|  | Mild | 44 (41.9%) | 8(18.2%) | 36(81.8%) |  |
|  | Severe | 9(8.6%) | 1(11.1%) | 8(88.9%) | 0.656 |
|  | HBV |  |  |  |  |
|  | - | 108(69.2%) | 35(32.4%) | 73(67.6%) |  |
|  | + | 48 (30.8%) | 9(18.7%) | 39(81.3%) | 0.080 |
|  | HCV |  |  |  |  |
|  | - | 131(84.0%) | 40(30.5%) | 91(69.5%) |  |
|  | + | 25(16.0%) | 4(16.0%) | 21(84.0%) | 0.139 |
|  | Alcohol |  |  |  |  |
|  | NO | 109(69.9%) | 28(25.7%) | 81(74.3%) |  |
|  | Alcohol consumption | 47(30.1%) | 16(34.0%) | 31(66.0%) | 0.287 |
|  | Liver fibrosis ishak score category |  |  |  |  |
|  | No Fibrosis | 29(32.6%) | 11(37.9%) | 18(62.1%) |  |
|  | Portal Fibrosis | 17(19.1%) | 5(29.4%) | 12(70.6%) |  |
|  | Fibrous Speta | 8(9.0%) | 2(25.0%) | 6(75.0%） |  |
|  | Nodular Formation and Incomplete Cirrhosis | 4(4.5%) | 1(25.0%) | 3(75.0%) |  |
|  | Established Cirrhosis | 31(34.8%) | 9(29.0%) | 12(71.0%) | 0.923 |
|  | Race Category |  |  |  |  |
|  | AMERICAN INDIAN OR ALASKA NATIVE | 67(44.7%) | 19(28.4%) | 48(71.6%) |  |
|  | ASIAN | 7(4.7%) | 2(28.6%) | 5(71.4%) |  |
|  | BLACK OR AFRICAN AMERICAN | 76(50.7%) | 23(30.3%) | 53(69.7%) | 0.968 |
|  | Gender |  |  |  |  |
|  | FEMALE | 55(35.3%) | 15(27.3%) | 40(72.7%) |  |
|  | MALE | 101(64.7%) | 29(28.7%) | 72(71.3%) | 0.849 |
|  | Vascular Invasion |  |  |  |  |
|  | NONE | 83(61.9%) | 24(28.9%) | 59(71.1%) |  |
|  | MICRO | 43(32.1%) | 14(32.6%) | 29(67.4%) |  |
|  | MACRO | 8(6.0%) | 2(25.0%) | 6(75.0%) | 0.872 |
| **Testing Cohort** | Age |  |  |  |  |
|  | <65 | 96(61.5%) | 16(16.7%) | 80(83.3%) |  |
|  | >65 | 60(38.5%) | 12(20.0%) | 48(80.0%) | 0.598 |
|  | M |  |  |  |  |
|  | M0 | 108(100%) | 22(20.4%) | 86(79.6%) |  |
|  | M1 | 0 |  |  |  |
|  | N |  |  |  |  |
|  | N0 | 104(97.2%) | 18(17.3%) | 86(82.7%) |  |
|  | N1 | 3(2.8%) | 2(66.7%) | 1(33.3%) | 0.031 |
|  | Stage |  |  |  |  |
|  | Stage I&II | 110(74.8%) | 19(17.3%) | 91(82.7%) |  |
|  | Stage III&IV | 37(25.2%) | 8(21.6%) | 29(78.4%) | 0.555 |
|  | T |  |  |  |  |
|  | T1&2 | 117(75.5%) | 21(17.9%) | 96(82.1%) |  |
|  | T3&4 | 38(24.5%) | 7(18.4%) | 31(81.6%) | 0.948 |
|  | Child pugh classification |  |  |  |  |
|  | A&B | 91(90.1%) | 14(15.4%) | 77(84.6%) |  |
|  | C&D | 10(9.9%) | 1(10.0%) | 9(90.0%) | 0.649 |
|  | Performance Status |  |  |  |  |
|  | Status 0 | 71(56.3%) | 7(9.9%) | 64(90.1%) |  |
|  | Status 1 | 35(27.8%) | 7(20.0%) | 28(80.0%) |  |
|  | Status 2 | 13(10.3%) | 5(38.5%) | 8(61.5%) |  |
|  | Status 3 | 7(5.6%) | 3(42.9%) | 4(57.1%) | 0.018 |
|  | Family History |  |  |  |  |
|  | NO | 84(61.8%) | 16(19.0%) | 68(81.0%) |  |
|  | YES | 52(38.2%) | 8(15.4%) | 44(84.6%) | 0.586 |
|  | Grade |  |  |  |  |
|  | G1&2 | 98(64.5%) | 13(13.3%) | 85(86.7%) |  |
|  | G3&4 | 54(35.5%) | 15(27.8%) | 39(72.2%) | 0.027 |
|  | Adjacent hepatic tissue inflammation |  |  |  |  |
|  | None | 54(51.9%) | 5(9.3%) | 49(90.7%) |  |
|  | Mild | 44(42.3%) | 11(25.0%) | 33(75.0%) |  |
|  | Severe | 6(5.8%) | 0(0%) | 6(100.0%) | 0.056 |
|  | HBV |  |  |  |  |
|  | - | 109(69.9%) | 22(20.2%) | 87(79.8%) |  |
|  | + | 47(30.1%) | 6(12.8%) | 41(87.2%) | 0.268 |
|  | HCV |  |  |  |  |
|  | - | 132(84.6%) | 25(18.9%) | 107(81.1%) |  |
|  | + | 24(15.4%) | 3(12.5%) | 21(87.5%) | 0.450 |
|  | Alcohol |  |  |  |  |
|  | NO | 100(64.1%) | 17(17.0%) | 83(83.0%) |  |
|  | Alcohol consumption | 56(35.9%) | 11(19.6%) | 45(80.4%) | 0.680 |
|  | Liver fibrosis ishak score category |  |  |  |  |
|  | No Fibrosis | 36(37.9%) | 6(16.7%) | 30(83.3%) |  |
|  | Portal Fibrosis | 8(8.4%) | 1(12.5%) | 7(87.5%) |  |
|  | Fibrous Speta | 18(18.9%) | 3(16.7%) | 15(83.3%) |  |
|  | Nodular Formation and Incomplete Cirrhosis | 4(4.2%) | 1(25.0%) | 3(75.0%) |  |
|  | Established Cirrhosis | 29(30.5%) | 6(20.7%) | 23(79.3%) | 0.973 |
|  | Race Category  AMERICAN INDIAN OR ALASKA NATIVE | 2(1.3%) | 0(0%) | 2(100%) |  |
|  | ASIAN | 71(46.4%) | 12(16.9%) | 59(83.1%) |  |
|  | BLACK OR AFRICAN AMERICAN | 8(5.2%) | 3(37.5%) | 5(62.5%) |  |
|  | WHITE | 72(47.1%) | 13(18.1%) | 59(81.9%) | 0.472 |
|  | Gender |  |  |  |  |
|  | FEMALE | 47(30.1%) | 8(17.0%) | 39(83.0%) |  |
|  | MALE | 109(69.9%) | 20(18.3%) | 89(81.7%) | 0.843 |
|  | Vascular Invasion |  |  |  |  |
|  | NONE | 87(71.8%) | 16(18.4%) | 71(81.6%) |  |
|  | MICRO | 39(23.4%) | 9(23.1%) | 30(76.9%) |  |
|  | MACRO | 8(4.8%) | 0(0%) | 8(100%) | 0.310 |
| **TCGA** | Age |  |  |  |  |
|  | <65 | 191(61.2%) | 45(23.6%) | `46(76.4%) |  |
|  | >65 | 121(38.8%) | 27(22.3%) | 94(77.7%) | 0.799 |
|  | M |  |  |  |  |
|  | M0 | 228(98.7%) | 54(23.7%) | 174(76.3%) |  |
|  | M1 | 3(1.3%) | 0(0%) | 3(100.0%) | 0.336 |
|  | N |  |  |  |  |
|  | N0 | 219(98.7%) | 50(22.8%) | 169(77.2%) |  |
|  | N1 | 3(1.3%) | 2(66.7%) | 1(33.3%) | 0.075 |
|  | Stage |  |  |  |  |
|  | Stage I&II | 217(74.3%) | 47(21.7%) | 170(78.3%) |  |
|  | Stage III&IV | 75(25.7%) | 20(26.7%) | 55(73.3%) | 0.374 |
|  | T |  |  |  |  |
|  | T1&2 | 231(74.8%) | 52(22.5%) | 179(77.5%) |  |
|  | T3&4 | 78(25.2%) | 20(25.6%) | 58(74.4%) | 0.572 |
|  | Child pugh classification |  |  |  |  |
|  | A&B | 192(90.1%) | 38(19.8%) | 154(80.2%) |  |
|  | C&D | 21(9.9%) | 1(4.8%) | 20(95.2%) | 0.091 |
|  | Performance Status |  |  |  |  |
|  | Status 0 | 145(56.4%) | 25(17.2%) | 120(82.8%) |  |
|  | Status 1 | 75(29.2%) | 16(21.3%) | 59(78.7%) |  |
|  | Status 2 | 24(9.3%) | 7(29.2%) | 17(70.8%) |  |
|  | Status 3  Status 4 | 12(4.7%)  1(0.4%) | 6(50.0%)  1(100%) | 6(50.0%)  0(0%) | 0.018 |
|  | Family History |  |  |  |  |
|  | NO | 178(65.2%) | 43(24.2%) | 135(75.8%) |  |
|  | YES | 95(34.8%) | 20(21.1%) | 75(78.9%) | 0.562 |
|  | Grade |  |  |  |  |
|  | G1&2 | 192(62.5%) | 36(18.8%) | 156(81.3%) |  |
|  | G3&4 | 115(37.5%) | 36(31.3%) | 79(68.7%) | 0.012 |
|  | Adjacent hepatic tissue inflammation |  |  |  |  |
|  | None | 106(50.7%) | 17(16.0%) | 89(84.0%) |  |
|  | Mild | 88(42.1%) | 19(21.6%) | 69(78.4%) |  |
|  | Severe | 15(7.2%) | 1(6.7%) | 14(93.3%) | 0.306 |
|  | HBV |  |  |  |  |
|  | - | 217(69.6%) | 57(26.3%) | 160(73.7%) |  |
|  | + | 955(30.4%) | 15(15.8%) | 80(84.2%) | 0.043 |
|  | HCV |  |  |  |  |
|  | - | 263(84.3%) | 65(24.7%) | 198(75.3%) |  |
|  | + | 49(15.7%) | 7(14.3%) | 42(85.7%) | 0.112 |
|  | Alcohol |  |  |  |  |
|  | NO | 209(67.0%) | 45(21.5%) | 164(78.5%) |  |
|  | Alcohol consumption | 103(33.0%) | 27(26.2%) | 76(73.8%) | 0.356 |
|  | Liver fibrosis ishak score category |  |  |  |  |
|  | No Fibrosis | 65(35.3%) | 17(26.2%) | 48(73.8%) |  |
|  | Portal Fibrosis | 25(13.6%) | 6(24.0%) | 19(76.0%) |  |
|  | Fibrous Speta | 26(14.1%) | 5(19.2%) | 21(80.8%) |  |
|  | Nodular Formation and Incomplete Cirrhosis | 8(4.3%) | 2(25.0%) | 6(75.0%) |  |
|  | Established Cirrhosis | 60(32.6%) | 15(25.0%) | 45(75.0%) | 0.974 |
|  | Race Category  AMERICAN INDIAN OR ALASKA NATIVE |  |  |  |  |
|  | ASIAN | 2(0.7%) | 0(0.0%) | 2(100%) |  |
|  | BLACK OR AFRICAN AMERICAN | 138(45.5%) | 31(22.5%) | 107(77.5%) |  |
|  | WHITE | 15(5.0%) | 5(33.3%) | 10(66.7%) |  |
|  | Gender | 148(48.8%) | 36(24.3%) | 112(75.7%) | 0.674 |
|  | FEMALE |  |  |  |  |
|  | MALE | 102(32.7%) | 23(22.5%) | 79(77.5%) |  |
|  | Vascular Invasion | 210(67.3%) | 49(23.3%) | 161(76.7%) | 0.877 |
|  | NONE |  |  |  |  |
|  | MICRO | 170(63.4%) | 40(23.5%） | 130(76.5%） |  |
|  | MACRO | 82(30.6%) | 23(28%) | 59(72%) |  |
|  | Race Category  AMERICAN INDIAN OR ALASKA NATIVE | 16(6.0%) | 2(12.5%) | 14(87.5%) | 0.388 |
| **GSE116674** | Age |  |  |  |  |
|  | <65 | 54(84.4%) | 16(29.6%) | 38(70.4%) |  |
|  | ≥65 | 10(15.6%) | 4(40.0%) | 6(60.0%) | 0.516 |
|  | Gender |  |  |  |  |
|  | FEMALE | 6(9.4%) | 1(16.7%) | 5(83.3%) |  |
|  | MALE | 58(90.6%) | 19(32.8%) | 39(67.2%) | 0.418 |
|  | Stage |  |  |  |  |
|  | Stage I&II | 53(82.8%) | 17(32.1%) | 36(67.9%) |  |
|  | Stage III&IV | 11(17.2%) | 3(27.3%) | 8(72.7%) | 0.754 |
|  | HBV  -  +  Alcohol  NO  YES  Smoke  NO  YES  Invasion  NO  YES | 17(26.6)  47(73.4)  51(79.7%)  13(20.3%)  32(50.8%)  31(49.2%)  35(54.7%)  29(45.3) | 5(29.4%)  15(31.9%)  18(35.3%)  2(15.4%)  9(28.1%)  10(32.3%)  10(28.6%)  10(34.5%) | 12(70.6%)  32(68.1%)  33(64.7%)  11(84.6%)  23(71.9%)  21(67.7%)  25(71.4%)  19(65.5%) | 0.849  0.167  0.721  0.612 |
