# Supplementary material for: A novel molecular-clinicopathologic nomogram to improve prognosis prediction of hepatocellular carcinoma
Source: Aging (Albany NY). 2020 Jun 30;12(13):12896–920. doi: 10.18632/aging.103350 (PMC7377850; doi:10.18632/aging.103350)
Supplement: Supplementary Table 11 [file aging-12-103350-s003..docx]

| **Supplementary Table 11. Correlations between risk score of the 14-lncRNA-based classifier with recurrence and clinicopathological characteristics in training cohort, test cohort, TCGA cohort and GEO cohort.** | | | | | |
| --- | --- | --- | --- | --- | --- |
| Clinicopathological variables | | Number of patients | High risk | Low risk | P value |
| Training Cohort | Age |  |  |  |  |
|  | <65 | 77(59.2%) | 31 (40.3%) | 46 (59.7%) |  |
|  | ≥65 | 53(40.8%) | 18 (34.0%) | 35 (66.0%) | 0.467 |
|  | M |  |  |  |  |
|  | M0 | 95(97.9%) | 37(38.9%) | 58(61.1%) |  |
|  | M1 | 2(2.1%) | 1(50%) | 1(50%) | 0.751 |
|  | N |  |  |  |  |
|  | N0 | 92(98.9%) | 35(38.0%) | 5(62%) |  |
|  | N1 | 1(1.1%) | 1(100%) | 0(0%) | 0.206 |
|  | Stage |  |  |  |  |
|  | Stage I&II | 99(81.1%) | 31(31.3%) | 68(68.7%) |  |
|  | Stage III&IV | 23(18.9%) | 13(56.5%) | 10(43.5%) | 0.023 |
|  | T |  |  |  |  |
|  | T1&2 | 103(79.8%) | 33(32.0%) | 70(68%) |  |
|  | T3&4 | 26(20.2%) | 16(61.5%) | 10(38.5%) | 0.006 |
|  | Child pugh classification |  |  |  |  |
|  | A&B | 87(90.6%) | 31(35.6%) | 56(64.4%) |  |
|  | C&D | 9(9.4%) | 0(0%) | 9(100%) | 0.030 |
|  | Performance Status |  |  |  |  |
|  | Status 0 | 73(66.4%) | 23(31.5%) | 50(68.5%) |  |
|  | Status 1 | 24(21.8%) | 8(33.3%) | 16(66.7%) |  |
|  | Status 2 | 9(8.2%) | 5(4.5%) | 4(3.6%) |  |
|  | Status 3 | 4(3.6%) | 4(3.6%) | 0(0%) | 0.026 |
|  | Family History |  |  |  |  |
|  | NO | 72(64.3%) | 30(41.7%) | 42(58.3) |  |
|  | YES | 40(35.7%) | 14(35%) | 26(65%) | 0.489 |
|  | Grade |  |  |  |  |
|  | G1&2 | 84(66.1%) | 29(34.5%) | 55(65.5%) |  |
|  | G3&4 | 43(33.9%) | 19(44.2%) | 24(55.8%) | 0.288 |
|  | Adjacent hepatic tissue inflammation |  |  |  |  |
|  | None | 43(50.0%) | 16(37.2%) | 27(62.8%) |  |
|  | Mild | 35(40.7%) | 9(25.7%) | 26(74.3%) |  |
|  | Severe | 8(9.3%) | 3(37.5%) | 5(62.5%) | 0.533 |
|  | HBV |  |  |  |  |
|  | - | 83(63.8%) | 36(43.4%) | 47(56.6%) |  |
|  | + | 47(36.2%) | 13(27.7%) | 34(72.3%) | 0.076 |
|  | HCV |  |  |  |  |
|  | - | 104(80.0%) | 37(35.6%) | 67(64.4%) |  |
|  | + | 26(20.0%) | 12(46.2%) | 14(53.8%) | 0.320 |
|  | Alcohol |  |  |  |  |
|  | NO | 88(67.7%) | 34(38.6%) | 54(61.4%) |  |
|  | Alcohol consumption | 42(32.3%) | 15(35.7%) | 27(64.3%) | 0.748 |
|  | Liver fibrosis ishak score category |  |  |  |  |
|  | No Fibrosis | 24(32%) | 7(29.2%) | 17(70.8%) |  |
|  | Portal Fibrosis | 11(14.7%) | 4(36.4%) | 7(63.6%) |  |
|  | Fibrous Speta | 16(21.3%) | 4(25.0%) | 12(75.0%） |  |
|  | Nodular Formation and Incomplete Cirrhosis | 3(4%) | 0(0.0%) | 3(100.0%) |  |
|  | Established Cirrhosis | 21(28%) | 9(42.9%) | 12(57.1%) | 0.542 |
|  | Race Category |  |  |  |  |
|  | AMERICAN INDIAN OR ALASKA NATIVE | 2(1.6%) | 0(0.0%) | 2(100.0%) |  |
|  | ASIAN | 64(50.0%) | 26(40.6%) | 38(59.4%) |  |
|  | BLACK OR AFRICAN AMERICAN | 1(0.8%) | 1(100.0%) | 0(0.0%) |  |
|  | WHITE | 61(47.7%) | 22(36.1%) | 39(30.5%) | 0.372 |
|  | Gender |  |  |  |  |
|  | FEMALE | 41(31.5%) | 15(36.6%) | 26(63.4%) |  |
|  | MALE | 89(68.5%) | 34(38.2%) | 55(61.8%) | 0.860 |
|  | Vascular Invasion |  |  |  |  |
|  | NONE | 64(58.2%) | 24(37.5%) | 40(62.5%) |  |
|  | MICRO | 38(34.5%) | 15(39.5%) | 23(60.5%) |  |
|  | MACRO | 8(7.3%) | 3(37.5%) | 5(62.5%) | 0.980 |
| Test Cohort | Age |  |  |  |  |
|  | <65 | 91(65.5%) | 40(44.0%) | 51(56%) |  |
|  | ≥65 | 48(34.5%) | 23(47.9%) | 25(52.1%) | 0.656 |
|  | M |  |  |  |  |
|  | M0 | 101(99.0%) | 41(40.6%) | 60(59.4%) |  |
|  | M1 | 1(100.0%) | 1(100.0%) | 0(0.0%) | 0.230 |
|  | N |  |  |  |  |
|  | N0 | 98(98.0%) | 41(41.8%) | 57(58.2%) |  |
|  | N1 | 2(2.0%) | 2(100.0%) | 0(0.0%) | 0.100 |
|  | Stage |  |  |  |  |
|  | Stage I&II | 90(69.8%) | 36(40.0%) | 54(60.0%) |  |
|  | Stage III&IV | 39(30.2%) | 21(53.8%) | 18(46.2%) | 0.146 |
|  | T |  |  |  |  |
|  | T1&2 | 100(73.0%) | 45(45.0%) | 55(55.0%) |  |
|  | T3&4 | 37(27.0%) | 18(48.6%) | 19(51.4%) | 0.704 |
|  | Child pugh classification |  |  |  |  |
|  | A&B | 81(92.0%) | 31(38.3%) | 50(61.7%) |  |
|  | C&D | 7(8.0%) | 5(71.4%) | 2(28.6%) | 0.087 |
|  | Performance Status |  |  |  |  |
|  | Status 0 | 63(54.8%) | 23(36.5%) | 40(63.5%) |  |
|  | Status 1 | 34(29.6%) | 16(47.1%) | 18(52.9) |  |
|  | Status 2 | 11(9.6%) | 6(54.5%) | 5(45.5%) |  |
|  | Status 3 | 7(6.1%) | 2(28.6%) | 5(71.4%) |  |
|  | Family History |  |  |  |  |
|  | NO | 78(63.9%) | 32(41.0%) | 46(59.0%) |  |
|  | YES | 44(36.1%) | 21(47.7%) | 23(52.3%) | 0.473 |
|  | Grade |  |  |  |  |
|  | G1&2 | 83(60.1%) | 30(36.1%) | 53(63.9%) |  |
|  | G3&4 | 55(39.9%) | 33(60.0%) | 22(40.0%) | 0.006 |
|  | Adjacent hepatic tissue inflammation |  |  |  |  |
|  | None | 48(50.0%) | 21(43.8%) | 27(56.3%) |  |
|  | Mild | 43(44.8%) | 19(44.2%) | 24(55.8%) |  |
|  | Severe | 5(5.2%) | 2(40.0%) | 3(60.0%) | 0.984 |
|  | HBV |  |  |  |  |
|  | - | 99(71.2%) | 53(53.5%) | 46(46.5%) |  |
|  | + | 40(28.8%) | 10(25.0%) | 30(75.0%) | 0.002 |
|  | HCV |  |  |  |  |
|  | - | 121(87.1%) | 54(44.6%) | 67(55.4%) |  |
|  | + | 18(12.9%) | 9(50.0%) | 9(50.0%) | 0.669 |
|  | Alcohol |  |  |  |  |
|  | NO | 88(63.3%) | 40(45.5%) | 48(54.5%) |  |
|  | Alcohol consumption | 51(36.7%) | 23(45.1%) | 28(54.9%) | 0.968 |
|  | Liver fibrosis ishak score category |  |  |  |  |
|  | No Fibrosis | 32(37.6%) | 13(40.6%) | 19(59.4%) |  |
|  | Portal Fibrosis | 8(9.4%) | 3(37.5%) | 5(62.5%) |  |
|  | Fibrous Speta | 10(11.8%) | 5(50.0%) | 5(50.0%) |  |
|  | Nodular Formation and Incomplete Cirrhosis | 2(2.4%) | 1(50.0%) | 1(50.0%) |  |
|  | Established Cirrhosis | 33(38.8%) | 13(39.4%) | 20(60.6%) | 0.976 |
|  | Race Category |  |  |  |  |
|  | ASIAN | 60(44.8%) | 30(50.0%) | 30(50%) |  |
|  | BLACK OR AFRICAN AMERICAN | 10(7.5%) | 5(50.0%) | 5(50.0%) |  |
|  | WHITE | 64(47.8%) | 26(40.6%) | 38(59.4%) |  |
|  | Gender |  |  |  |  |
|  | FEMALE | 51(36.7%) | 26(51.0%) | 25(49.0%) |  |
|  | MALE | 88(63.3%) | 37(42.0%) | 51(36.7%) | 0.308 |
|  | Vascular Invasion |  |  |  |  |
|  | NONE | 89(71.8%) | 42(47.2%) | 47(52.8%) |  |
|  | MICRO | 29(23.4%) | 11(37.9%) | 18(62.1%) |  |
|  | MACRO | 6(4.8%) | 4(66.7%) | 2(33.3%) | 0.398 |
| TCGA Cohort | Age |  |  |  |  |
|  | <65 | 168(62.5%) | 71(42.3%) | 97(57.7%) |  |
|  | ≥65 | 101(37.5%) | 41(40.6%) | 60(59.4%) | 0.788 |
|  | M |  |  |  |  |
|  | M0 | 196(98.5%) | 78(39.8%) | 118(60.2%) |  |
|  | M1 | 3(1.5%) | 2(66.7%) | 1(33.3%) | 0.346 |
|  | N |  |  |  |  |
|  | N0 | 190(98.4%) | 76(40.0%) | 114(60.0%) |  |
|  | N1 | 3(1.6%) | 3(100.0%) | 0(0.0%) | 0.036 |
|  | Stage |  |  |  |  |
|  | Stage I&II | 189(75.3%) | 67(35.4%) | 122(64.6%) |  |
|  | Stage III&IV | 62(24.7%) | 34(54.8%) | 28(45.2%) | 0.007 |
|  | T |  |  |  |  |
|  | T1&2 | 203(76.3%) | 78(38.4%) | 125(61.6%) |  |
|  | T3&4 | 63(23.7%) | 34(54.0%) | 29(46.0%) | 0.029 |
|  | Child pugh classification |  |  |  |  |
|  | A&B | 168(91.3%) | 62(36.9%) | 106(63.1%) |  |
|  | C&D | 16(8.7%) | 5(31.3%) | 11(68.8%) | 0.653 |
|  | Performance Status |  |  |  |  |
|  | Status 0 | 136(60.4%) | 46(33.8%) | 90(66.2%) |  |
|  | Status 1 | 58(25.8%) | 24(41.4%) | 34(58.6%) |  |
|  | Status 2 | 20(8.9%) | 11(55.0%) | 9(45.0%) |  |
|  | Status 3 | 11(4.9%) | 6(54.5%) | 5(45.5%) | 0.176 |
|  | Family History |  |  |  |  |
|  | NO | 150(64.1%) | 62(41.3%) | 88(58.7%) |  |
|  | YES | 84(35.9%) | 35(41.7%) | 49(58.3%) | 0.960 |
|  | Grade |  |  |  |  |
|  | G1&2 | 167(63.0%) | 59(35.3%) | 108(64.7%) |  |
|  | G3&4 | 98(37.0%) | 52(53.1%) | 46(46.9%) | 0.005 |
|  | Adjacent hepatic tissue inflammation |  |  |  |  |
|  | None | 91(50.0%) | 37(40.7%) | 54(59.3%) |  |
|  | Mild | 78(42.9%) | 28(35.9%) | 50(64.1%) |  |
|  | Severe | 13(7.1%) | 5(38.5%) | 8(61.5%) | 0.818 |
|  | HBV |  |  |  |  |
|  | - | 182(67.7%) | 89(48.9%) | 93(51.1%) |  |
|  | + | 87(32.3%) | 23(26.4%) | 64(73.6%) | 0.001 |
|  | HCV |  |  |  |  |
|  | - | 225(83.6%) | 91(40.4%) | 134(59.6%) |  |
|  | + | 44(16.4%) | 21(47.7%) | 23(52.3%) | 0.370 |
|  | Alcohol |  |  |  |  |
|  | NO | 176(65.4%) | 74(42.0%) | 102(58.0%) |  |
|  | Alcohol consumption | 93(34.6%) | 38(40.9%) | 55(59.1%) | 0.851 |
|  | Liver fibrosis ishak score category |  |  |  |  |
|  | No Fibrosis | 56(35.0%) | 20(35.7%) | 36(64.3%) |  |
|  | Portal Fibrosis | 19(11.9%) | 7(36.8%) | 12(63.2%) |  |
|  | Fibrous Speta | 26(16.3%) | 9(34.6%) | 17(65.4%) |  |
|  | Nodular Formation and Incomplete Cirrhosis | 5(3.1%) | 1(20.0%) | 4(80.0%) |  |
|  | Established Cirrhosis | 54(33.8%) | 22(40.7%) | 32(59.3%) | 0.902 |
|  | Race Category |  |  |  |  |
|  | AMERICAN INDIAN OR ALASKA NATIVE | 2(8%) | 0(0.0%) | 2(0.8%) |  |
|  | ASIAN | 124(47.3%) | 56(45.2%) | 68(54.8%) |  |
|  | BLACK OR AFRICAN AMERICAN | 11(4.2%) | 6(54.5%) | 5(45.4%) |  |
|  | WHITE | 125(47.7%) | 48(38.4%) | 77(61.6%) | 0.343 |
|  | Gender |  |  |  |  |
|  | FEMALE | 92(34.2%) | 41(44.6%) | 51(55.4%) |  |
|  | MALE | 177(65.8%) | 71(40.1%) | 106(59.9%) | 0.482 |
|  | Vascular Invasion |  |  |  |  |
|  | NONE | 153(65.4%) | 66(43.1%） | 87(56.9%） |  |
|  | MICRO | 67(28.6%) | 26(38.8%) | 41(61.2%) |  |
|  | MACRO | 14(6.0%) | 7(50.0%) | 7(50.0%) | 0.689 |
| GSE76427 Cohort | Age |  |  |  |  |
|  | <65 | 45(55.6%) | 19(42.2%) | 26(57.8%) |  |
|  | ≥65 | 36(44.4%) | 15(41.7%) | 21(58.3%) | 0.960 |
|  | Gender |  |  |  |  |
|  | FEMALE | 11(13.6%) | 7(63.6%) | 4(36.4%) |  |
|  | MALE | 70(86.4%) | 27(38.6%) | 43(61.4%) | 0.117 |
|  | Stage |  |  |  |  |
|  | Stage I&II | 64(79.0%) | 25(39.1%) | 39(60.9%) |  |
|  | Stage III&IV | 17(21.0%) | 9(52.9%) | 8(47.1%) | 0.303 |
|  |  |  |  |  |  |
