# Supplementary material for: A novel molecular-clinicopathologic nomogram to improve prognosis prediction of hepatocellular carcinoma
Source: Aging (Albany NY). 2020 Jun 30;12(13):12896–920. doi: 10.18632/aging.103350 (PMC7377850; doi:10.18632/aging.103350)
Supplement: Supplementary Table 12 [file aging-12-103350-s006..docx]

| **Supplementary Table 12. Univariate and multivariate COX analyses of the lncRNA-based classifier for OS.** | | | | | | | |
| --- | --- | --- | --- | --- | --- | --- | --- |
| Prognostic parameter | Univariate analysis | | |  | Multivariate analysis | | |
|  | HR | 95% CI | P value |  | HR | 95% CI | P value |
| **Training Cohort** |  |  |  |  |  |  |  |
| RiskScore | 7.608 | 4.143-13.971 | **<0.001** |  | 24.461 | 8.192-73.039 | **<0.001** |
| Age | 0.867 | 0.503-1.495 | 0.608 |  |  |  |  |
| M | 4.373 | 1.335-14.326 | **0.015** |  | 5.704 | 1.313-24.777 | **0.020** |
| N  Stage | 3.172 | 1.805-5.574 | **<0.001** |  | 1.616 | 0.653-3.997 | 0.299 |
| T classification | 3.035 | 1.782-5.170 | **<0.001** |  | 1.616 | 0.653-3.997 | 0.299 |
| Bilirubin | 1.143 | 0.877-1.488 | 0.323 |  |  |  |  |
| Child pugh classification  Performance Status  Family History  Fraction Genome Altered  Grade  Adjacent hepatic tissue inflammation  HBV  HCV  Alcohol  Liver fibrosis ishak score category  Mutation Count  Platelet count  Race Category  Albumin  Gender  Vascular Invasion  BMI  AFP | 0.744  2.132  1.023  1.848  0.999  1.159  0.509  0.817  1.235  0.930  1.001  1.000  1.168  0.999  0.713  0.802  1.022  0.987 | 0.873-6.081  1.539-2.952  0.566-1.849  0.473-7.217  0.577-1.730  0.667-2.011  0.263-0.988  0.369-1.811  0.707-2.157  0.748-1.156  0.999-1.003  1.000-1.000  0.881-1.548  0.994-1.004  0.412-1.234  0.486-1.326  1.002-1.043  0.924-1.054 | 0.092  **<0.001**  0.939  0.377  0.999  0.601  **0.046**  0.619  0.459  0.513  0.452  0.357  0.279  0.740  0.227  0.391  **0.035**  0.689 |  | 1.748  1.041    1.093  1.000 | 1.154-2.650  0.433-2.504    0.564-2.120  0.944-1.060 | **0.008**  0.929    0.792  0.992 |
| **Test Cohort** |  |  |  |  |  |  |  |
| RiskScore | 3.349 | 1.727-6.496 | **<0.001** |  | 11.785 | 3.138-44.262 | **<0.001** |
| Age | 1.348 | 0.770-2.362 | 0.296 |  |  |  |  |
| M |  |  |  |  |  |  |  |
| N  Stage | 2.892  2.467 | 0.674-12.406  1.328-4.581 | 0.153  **0.004** |  | 0.139 | 0.012-1615 | 0.115 |
| T classification | 2.642 | 1.476-4.728 | **0.001** |  | 15.232 | 1.176-197.37 | **0.037** |
| Bilirubin | 0.901 | 0.679-1.197 | 0.473 |  |  |  |  |
| Child pugh classification  Performance Status  Family History  Fraction Genome Altered  Grade  Adjacent hepatic tissue inflammation  HBV  HCV  Alcohol  Liver fibrosis ishak score category  Mutation Count  Platelet count  Race Category  Albumin  Gender  Vascular Invasion  BMI  AFP | 1.900  3.649  1.236  26.805  1.168  1.033  0.156  1.698  1.174  0.913  1.001  1.000  0.874  1.032  1.238  1.194  1.011  1.093 | 0.613-5.894  2.391-5.571  0.690-2.215  5.613-128.009  0.639-2.133  0.557-1.917  0.056-0.436  0.814-3.544  0.650-2.123  0.712-1.171  0.999-1.002  1.000-1.000  0.663-1.153  0.980-1.088  0.665-2.304  0.750-1.900  0.973-1.051  1.016-1.175 | 0.266  **<0.001**  0.476  **<0.001**  0.614  0.918  **<0.001**  0.158  0.595  0.475  0.381  0.160  0.340  0.231  0.501  0.456  0.565  **0.018** |  | 2.983  22.162  0.186  1.549  1.004 | 1.734-5.133  1.518-323.581  0.037-0.943  0.742-3.234  0.873-1.155 | **<0.001**  **0.024**  **0.042**  0.244  0.951 |
| **TCGA Cohort** |  |  |  |  |  |  |  |
| RiskScore | 5.098 | 3.289-7.900 | **<0.001** |  | 15.483 | 6.149-38.989 | **<0.001** |
| Age | 1.057 | 0.717-1.558 | 0.781 |  |  |  |  |
| M | 5.141 | 1.603-16.486 | **0.006** |  | 7.703 | 1.603-37.021 | **0.011** |
| N  Stage | 2.561  2.849 | 0.623-10.530  1.885-4.305 | 0.192  **<0.001** |  | 1.701 | 0.646-4.482 | 0.282 |
| T classification | 2.855 | 1.934-4.214 | **<0.001** |  |  |  |  |
| Bilirubin | 0.970 | 0.829-1.134 | 0.699 |  |  |  |  |
| Child pugh classification  Performance Status  Family History  Fraction Genome Altered  Grade  Adjacent hepatic tissue inflammation  HBV  HCV  Alcohol  Liver fibrosis ishak score category  Mutation Count  Platelet count  Race Category  Albumin  Gender  Vascular Invasion  BMI  AFP  **GSE116674 Cohort**  RiskScore  Age  Gender  Stage  HBV  Alcohol  Smoke  Vascular Invasion | 2.112  2.609  1.161  4.219  1.064  1.087  0.318  1.151  1.206  0.941  1.001  1.000  1.014  0.999  0.896  0.990  1.021  1.017  2.941  1.303  3.320  1.576  0.783  1.099  1.459  3.879 | 1.025-4.350  2.033-3.348  0.773-1.744  1.583-11.245  0.710-1.593  0.722-1.637  0.184-0.550  0.674-1.968  0.805-1.807  0.803-1.104  0.999-1.002  1.000-1.000  0.833-1.235  0.995-1.003  0.597-1.345  0.704-1.392  1.002-1.041  0.971-1.066  1.004-8.619  0.491-3.453  0.450-24.511  0.636-3.909  0.342-1.794  0.443-2.727  0.673-3.163  1.723-8.729 | 0.043  **<0.001**  0.473  **0.004**  0.764  0.689  **<0.001**  0.606  0.364  0.457  0.320  **0.097**  0.888  0.677  0.597  0.955  **0.034**  0.478  **0.049**  0.595  0.239  0.326  0.564  0.839  0.339  **0.001** |  | 1.670  2.589  0.308  0.805  1.410  0.998  3.343  3.869 | 0.525-5.307  1.355-4.947  0.026-3.598  0.352-1.841  0.841-2.364  0.953-1.045  0.986-11.334  1.719-8.708 | 0.385  **0.004**  0.348  0.607  0.192  0.927  0.053  **0.001** |
| HR, Hazard ratio; CI, confidence interval; lncRNA, long non-coding RNA. | | | | | | | |
